# Effects of site-directed mutagenesis of *mglA *on motility and swarming of *Myxococcus xanthus*

**DOI:** 10.1186/1471-2180-10-295

**Published:** 2010-11-18

**Authors:** Sarah A Fremgen, Neal S Burke, Patricia L Hartzell

**Affiliations:** 1 Department of Microbiology, Molecular Biology and Biochemistry, 142 Life Science, University of Idaho, Moscow ID 83844-3052, USA; 2 Current Address: Department of Veterinary Medicine, Washington State University, Pullman, WA 99164 USA

## Abstract

**Background:**

The *mglA *gene from the bacterium *Myxococcus xanthus *encodes a 22kDa protein related to the Ras superfamily of monomeric GTPases. MglA is required for the normal function of A-motility (adventurous), S-motility (social), fruiting body morphogenesis, and sporulation. MglA and its homologs differ from all eukaryotic and other prokaryotic GTPases because they have a threonine (Thr78) in place of the highly conserved aspartate residue of the consensus PM3 (phosphate-magnesium binding) region. To identify residues critical for MglA function or potential protein interactions, and explore the function of Thr78, the phenotypes of 18 *mglA *mutants were characterized.

**Results:**

Nine mutants, with mutations predicted to alter residues that bind the guanine base or coordinate magnesium, did not produce detectable MglA. As expected, these mutants were mot^- ^dev^- ^because MglA is essential for these processes. Of the remaining nine mutants, seven showed a wild-type distribution pattern for MglA but fell into two categories with regard to function. Five of the seven mutants exhibited mild phenotypes, but two mutants, T78D and P80A, abolished motility and development. The localization pattern of MglA was abolished in two mutants that were mot^- ^spo^- ^and dev^-^. These two mutants were predicted to alter surface residues at Asp52 and Thr54, which suggests that these residues are critical for proper localization and may define a protein interaction site. Improving the consensus match with Ras at Thr78 abolished function of MglA. Only the conservative serine substitution was tolerated at this position. Merodiploid constructs revealed that a subset of alleles, including *mglA*D52A, were dominant and also illustrated that changing the balance of MglA and its co-transcribed partner, MglB, affects A-motility.

**Conclusion:**

Our results suggest that GTP binding is critical for stability of MglA because MglA does not accumulate in mutants that cannot bind GTP. The threonine in PM3 of MglA proteins represents a novel modification of the highly conserved GTPase consensus at this position. The requirement for a hydroxyl group at this position may indicate that MglA is subject to modification under certain conditions. Proper localization of MglA is critical for both motility and development and likely involves protein interactions mediated by residues Asp52 and Thr54.

## Background

The Gram-negative soil bacterium *Myxococcus xanthus *is a model prokaryote for understanding the complexity of intercellular interactions that occur during multicellular development. When nutrients are limiting, groups of (>10^5^) *M. xanthus *cells can aggregate and assemble fruiting bodies. Inside fruiting bodies, cells differentiate to form resting spores which are resistant to heat, ultraviolet light, and desiccation [1]. Both the aggregation of cells during the morphogenesis of fruiting bodies and the differentiation of heat-resistant spores are dependent on subsets of genes involved in the ability of *M. xanthus *to glide over surfaces using two different mechanisms of locomotion, A-gliding and S-gliding.

Gliding does not depend on flagella. A-gliding depends on the functions of more than 30 different genes, which encode products that enable individual cell movement by a mechanism that may involve secretion of a polyelectrolyte [2] or motors that exist at focal adhesion sites [3,4]. S-gliding depends on the functions of more than 80 different genes, which encode proteins for the synthesis, regulation, and function of type IVpili (Tfp) [5], lipopolysaccharide (LPS) [6] and exopolysaccharide (EPS) [7-10]. Strains with mutations in an A gene are motile because they retain S-motility, yet they form colonies that are smaller than the wild-type (WT). Conversely, strains with mutations in an S-motility gene are motile because they retain A-motility yet they also form colonies that are smaller than the WT. A^-^S^- ^double mutants form colonies that lack flares at their edges, are unable to swarm (srm^-^) and are nonmotile (mot^-^) when viewed by time-lapse microscopy on 1.5% agar.

*mglA *mutants produce colonies with smooth edges that are identical to colonies of the A^-^S^- ^double mutants. They are described as nonmotile because they make no net movement, but when viewed by time lapse microscopy on the edge of a swarm, a few cells can be seen to reverse direction frequently [11]. The decreased efficiency of swarming outward from a central location may be due to a lack of coordination of the A and S-gliding motors by MglA.

The *mglA *gene encodes a 22 kD protein similar in sequence to members of the Ras (p21) superfamily of monomeric GTPases [12]. Some of the defects caused by an *mglA *deletion mutation can be complemented by the expression of the yeast GTPase, Sar1p, in place of *mglA *[12]. A Sar1p mutant that is unable to hydrolyze GTP fails to complement the *mglA *mutant, suggesting that GTPase activity is critical for MglA function. Like Sar1p, MglA has consensus motifs for GTP binding and hydrolysis that are conserved among members of the small GTPases [13]. Three of these regions contain residues that make contact with the Mg^2+ ^cofactor and ß and γ phosphates of GTP, and are called the PM (phosphate-magnesium binding) regions, and two of these regions are involved in specific contacts with the guanine ring, and are called the G regions [14]. An alternative convention labels the conserved motifs as G1 through G5 [15,16].

The MglA sequence contains the PM1 region (or "P loop") _19_GxxxxGKT_26_, which matches the consensus sequence, GxxxxGKT/S for small GTPases. A single conserved Thr defines PM2, for which several candidates exist in MglA between PM1 and PM3. The consensus sequence of PM3 is DxxGQ/T. Here MglA differs from consensus because the corresponding region of MglA, _78_TxxGQ_82_, contains a threonine instead of an aspartate residue [12]. Additionally, MglA contains identifiable motifs for guanine specificity. G1 is a conserved phenylalanine or tyrosine and G2 has the consensus N/TKxD. MglA has candidates for G1 in Phe 56, Phe 57 or Phe59. G2 makes critical interactions with the nucleotide base with the Asp side chain conferring specificity for guanine. The sequence _141_NKRD_144 _of MglA matches the G2 consensus, N/TKxD. We have not identified a candidate region for the G3 consensus motif in part because the side-chains of G3 in Ras assist in binding rather than interact directly with the nucleotide [13]. MglA lacks any identifiable motif for lipid modification such as palmitoylation or farnesylation seen with many eukaryotic Ras-like proteins.

Four recent studies confirmed *in vitro *GTPase activity of MglA from *M. xanthus *[4,17,18] and the thermophilic bacterium *Thermus thermophilus *[19]. Experiments in our laboratory using refolded purified MglA determined a hydrolysis rate of 1.224 h^-1 ^for MglA using a direct assay [17], similar to the intrinsic rate of Ras, as well as other bacterial GTPases, such as Era [20,21]. Surprisingly, hydrolysis rates of 40 s^-1 ^were observed for MglA using a coupled enzyme assay [4], which is consistent with the rates given for Ras stimulated by a GAP protein (19 s^-1^) [21]. Although not specified by the authors, it is possible that a stimulating component may have co-purified and stimulated these remarkable rates of GTPase activity, which are >2000 higher than any known bacterial GTPase. Zhang *et al*. reported that they derived similar rates [18]. Leonardy *et al*. reported hydrolysis rates of 0.32 h^-1 ^for purified MglA from *Thermus thermophilus*. The lower hydrolysis rate for the *Thermus *enzyme might be attributed to the fact that these assays were performed at 25°C, which is likely suboptimal for an enzyme from a hyperthermophile. Addition of stoichiometric amounts of *T. thermophilus *MglB has been reported to stimulate hydrolysis, inferring that MglB might be responsible for stimulation of GTP hydrolysis by MglA [19].

In this paper, we describe the phenotypes of a collection of *mglA *mutants that target consensus motifs or surface residues. Previous random mutagenesis of *mglA *revealed that several residues were critical for proper expression of the MglA protein. Mutants such as *mgl7*, which changed a Cys to a Phe in what is predicted to be PM1, failed to express detectable MglA whereas *mgl11*, which altered a residue in the PM3 region, did not adversely affect MglA expression [22]. We engineered mutations that affect residues critical for GTP binding and found that they had a severe effect on gliding because, in many cases, these mutants failed to produce stable MglA protein, echoing the earlier observations of Stephens *et al*. A subset of mutations affected swarming on 0.3% agar to a greater extent than swarming on 1.5% agar. Two mutations (one in a predicted surface residue and one involving restoration of a conserved motif) inhibited one or both motility systems in a dominant fashion. The results of this phenotypic analysis demonstrate that residues predicted to be essential for GTP binding and hydrolysis are critical for the functions of MglA in motility and development.

## Results and Discussion

### Model of the structure of MglA and alignment

MglA is a 21,999 Da protein [23] that shares identity (25.9%) and similarity (43.7%) with Harvey Ras (Harvey rat sarcoma viral oncogene homolog) also called Ha-Ras or p21-Ras, [Genbank:NP_005334.1] which is a well-characterized member of the Ras superfamily of monomeric GTPases found in eukaryotes. MglA shares 29% identity and 43% similarity with Sar1p from *Saccharomyces cerevisiae*, the GTPase that complements an *mglA *deletion [12], and 30% identity, 49% similarity with ADP-ribosylation factor-like protein 8 from *Dictyostelium discoideum *AX4.

**Figure 1:**
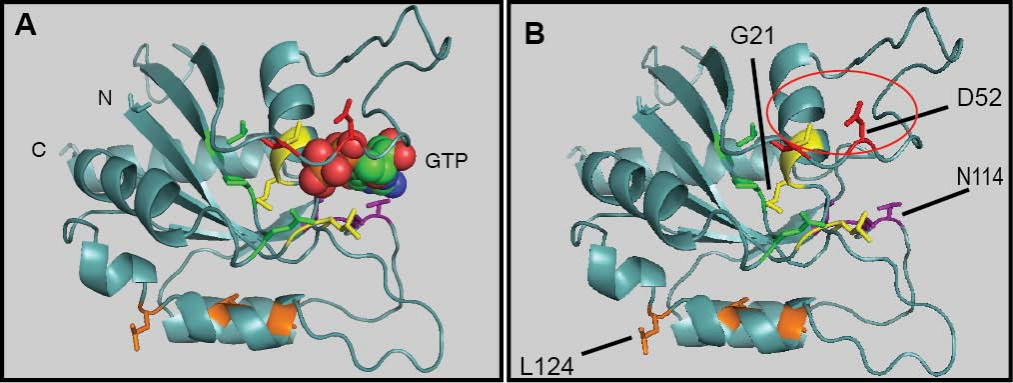
**A. *In silico *model of MglA with GPPNHP in the predicted active site; B. MglA model without docked nucleotide**. A three-dimensional representation of MglA was constructed with SWISS-MODEL using the crystal structure of Sar1p as a template [24-26] and the result is shown here as generated by PyMOL [27]. All mutations made in MglA were between residues 18 and 145. In both panels, targeted residues are colored as follows: P-loop (PM1), yellow; PM3, green; D52/T54, red; G2 motif, purple; leucine rich repeat (LRR), orange. Thr78 corresponds to the conserved aspartate residue characteristic of the Ras-superfamily, and is located at the end of the α-helix shown in green. Side-chains are shown for residues that were targets of study through site-directed mutagenesis. A: A GTP analog was docked with MglA to identify residues in or near the active site that might directly interact with either the guanine base or the phosphates. B: The MglA apoenzyme is shown with residues indicated. G21 denotes the location of the PM1 region, the N114 residue shown is in the G2 motif. Both D52A and L124 are predicted surface residues on opposite faces of the protein.

A three-dimensional model for MglA was constructed to identify residues that may be involved in protein-protein interactions and to examine ways in which MglA might deviate from other GTPases. While attempts to grow crystals with purified homogeneous MglA have not been successful, the homology between MglA and GTPases with previously derived crystal structure templates enabled us to model MglA using the SWISS-MODEL program [24-26]. The *in silico *structure of MglA was used to generate a 3-D molecular model that could be manipulated in PyMOL [27]. The predicted structure of MglA based on the Sar1p protein from *S. cerevisiae *(PDB ID 2QTV chain B), is shown in Figure 1. Alignment of MglA with the template sequence Sar1p allows for all conserved motifs to be correctly aligned with those in MglA, preserving the PM1 and PM3 regions.

As the crystal structure of the Sar1p template lacks a portion of the N-terminus and begins with residue 23 of the predicted peptide, our MglA model also lacks a portion of the N-terminus and begins with Asn12. The Sar1p template likewise lacks a C-terminal portion of the protein, and the best alignment was made possible by a truncation of MglA as well. Hence, the MglA model ends with Lys185, which truncates ten residues of MglA. Using PyMOL's alignment with least root mean square deviation (RMSD) of this model with the crystal structure of Sar1p containing GTP, we were able to determine the approximate position where GTP would bind to MglA. This is shown in Figure 1A as a space-filling molecule.

The portion of MglA which contains the predicted surface residues D52 and T54 as circled in Figure 1B was identified as one of our candidate sites for protein-protein interaction and is referred to as the recruitment interface. We additionally constructed an overlaid diagram with both the MglA model and the known Ras crystal structure to identify if there were any locations that showed structural differences of import. Ras is illustrated in yellow, while MglA is displayed in red in the cartoon representation of Additional file 1: FigureS1MglARasoverlay. The MglA model contains a large loop of 13 amino acids that does not align with Ras, a phenomenon observed in other GTPases [28]. We have termed this loop the M-loop as it appears to be distinct from those observed in other GTPases.

### Motility, swarming, and development capabilities of MglA mutants were analyzed

*M. xanthus *strain DK6204 carries a deletion within the *mglBA *operon and is unable to swarm [23]. All *mglA *modifications were constructed on a DNA fragment that is necessary and sufficient to fully complement the motility and development defects of DK6204 (Δ*mglBA*) when integrated at the Mx8 attachment site or at the normal chromosomal site. For the studies presented here, all plasmids were electroporated into the Δ*mglBA *deletion strain DK6204 and Kan^R ^clones arose from recombination between *mgl *promoter on the plasmid and the *mgl *promoter that exists on the chromosome in DK6204. All complementing strains examined in this study were found to grow vegetatively with a doubling time comparable to the DK1622 (WT), DK6204 (Δ*mglBA *parent), and MxH2419 (DK6204::pKD100).

**Figure 2:**
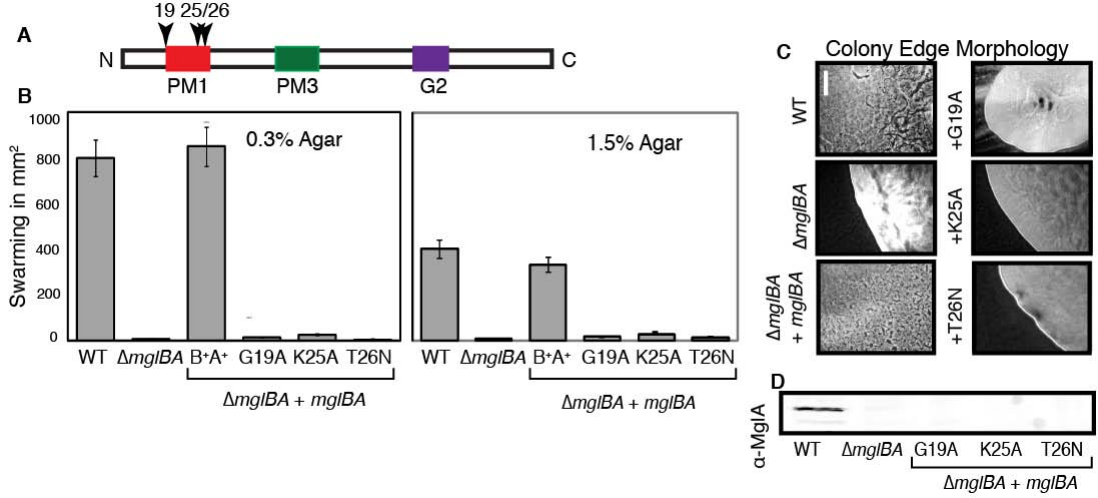
**Mutants in the P-loop fail to complement the motility defect of Δ*mglBA***. Mutations were generated in conserved residues Gly19 (MxH2445), Lys25 (MxH2430) and Thr26 (MxH2410) that define the γ-phosphate interacting region of MglA (P-loop). A. Graphic representation of the MglA protein, showing the relative position of PM1 (dark box). Residues mutated are indicated with an arrow head. B. (upper) Relative swarming of each strain on 1.5% CTPM agar; (lower) relative swarming of each strain on 0.3% CTPM agar. The WT *M. xanthus *strain DK1622 and Δ*mglBA *strain DK6204 are shown as the first and second bars respectively. The third bar (B^+^A^+^) shows the complemented control MxH2419 (Δ*mglBA*+pKD100). C. Colony edge morphology of isolated colonies on 1.5% CTPM agar at 100× magnification. Bar = 25 μm. D. Immunoblot showing production of MglA in each strain. PVDF membranes were probed with α-MglA (1:1000) and goat α-rabbit IgG tagged with Alexa Fluor 800 (1:2500).

The mutants were assayed for ability to swarm, A- and S-gliding characteristics at the colony edge, gliding rates and reversal frequency. Swarm data for the WT and Δ*mglBA *strains are represented by the first two bars of Figure 2B. WT displayed robust swarming on 1.5% (403 ± 25 mm^2^) and 0.3% (820 ± 66 mm^2^) agar. In contrast, swarming of the Δ*mglBA *strain was less than 2% of the WT. Addition of plasmid pKD100 (*mglBA^+^*) to DK6204 yielded MxH2419, which exhibited WT-like motility and development. Swarming of MxH2419 on 1.5% and 0.3% agar was 90 ± 9% and 100 ± 12% that of the WT, respectively. These data are presented in all swarm assay figures. For comparison, the phenotypes (swarming, gliding rates, and reversal frequency) of all complementing strains will be presented as a percentage of MxH2419, the reference control strain. The localization of MglA in cells gliding on agar and in methylcellulose is quite distinct [17] and we considered that certain MglA mutations might yield a phenotype if the ability of an MglA to interact with protein partners was affected. Hence, we assayed the localization of MglA in mutant strains using immunofluorescence as described in Methods. Localization patterns for each strain are shown in one common figure and are discussed in each section below.

### Mutations in the conserved PM1 consensus involved in GTP hydrolysis affect stability of MglA

The P-loop (PM1) is involved in hydrolysis of GTP in ATPases and GTPases. Mutations in PM1 were engineered to determine if residues known to be involved in GTP hydrolysis are needed for MglA activity. The corresponding region of MglA is previously shown in Figure 1, highlighted in yellow and begins with Gly19 in a random coil region and ends with Thr26 at the beginning of an α-helix. A linear diagram of MglA, shown in Figure 2A, indicates the location of the PM1 region. Three residues, Gly19, Lys25 and Thr26 that are conserved in the PM1 region of GTPases (GXXXXGKS/T), were targeted for mutagenesis. Residues Gly19 and Lys25 were substituted with alanine while Thr26 was substituted with asparagine using overlap PCR [29] to generate G19A, K25A and T26N. The T26N substitution was modeled after the dominant negative mutant of p21 Ras, which abolishes the ability of Ras-like proteins to properly coordinate magnesium and decreases affinity of Ras for GTP [30,31].

As shown in Figure 2B, addition of mutant alleles to the deletion strain failed to restore swarming to wild type levels. Swarming of G19A, K25A, and T26N was 4.9%, 7.9%, and 4.6% respectively on 1.5% agar and 1.3%, 2.7%, and 0.5% on 0.3% agar respectively compared to the control.

Swarming assays measure the ability of cells at high density to swarm over different surfaces but do not reveal information about specific motility behaviors. To examine the ability of individual cells to glide and reverse, time-lapse microscopy of cells at low density on 1.5% CTPM agarose was used. No single-cell movement was visible for G19A, K25A or T26N on 1.5% agarose identical to the behavior for the nonmotile Δ*mglBA *strain. In contrast, the control strain (MxH2419) moved at 2.1 ± 1.7 μm/min and reversed once every 14.8 min. Although a frequency of one reversal every 7.5 min has been previously published by Blackhart and Zusman for *M. xanthus *strain DZF1 [32], we hypothesize that differences in strains (DK1622 *vs*. DZF1 and DZ2), nutritional conditions, and preparation of samples may account for a difference in reversal frequency. Our preparations included twice the amount of Casitone in the agarose surface as previously published studies [32].

Software for tracking large numbers of cells works well at low cell density because cells are well isolated. This poses a problem to track cells using S-motility because close cell contact is necessary to stimulate retraction of pili. However, methylcellulose (MC) has been shown to serve as a substitute for cell-cell contact [33]. Therefore, to quantify S-motility of the *mgl *mutants, videomicroscopy of cells in 0.5% MC and CTPM was used. Under these conditions, WT cells reversed every 15.6 min on average and moved with an average speed of 4.8 μm/min (Additional file 2: Movie WT). PM1 mutants moved at speeds less than 50% of the control in MC (Table 1) and many of the cells exhibited an oscillating motion, a phenotype additionally observed in the Δ*mglBA *deletion parent in methylcellulose only (Additional file 3: Movie mglBA). The phenotype of the T26N strain MxH2410 (Additional file 4: Movie 3) is representative of the PM1 mutants, where 96% of the cells oscillate in methylcellulose. For reference, Additional file 5: Movie 4 depicts a strain that has lost both A and S motility through defects in the respective motors in the form of a *aglZ^- ^pilA^- ^*double mutant, showing that this behavior is not the result of Brownian motion.

**Table 1:** Comparison of Gliding Rates and Sporulation for mgl mutants

		Gliding on		Sporulation
		**A-motility^a^**	**S-motility^b^**	**Percent of WT^c^**

**Strain**	**Genotype**	**Average Speed in μm/min (Minutes per reversal)**		

WT	DK1622	2.6 (20.7)	4.8 (15.6)	100 ± 20

Δ*mglBA*	DK6204	NM	1.9 (10.3)	< 0.01

Δ*mglBA*+*mglBA^+^*	MxH2419	2.1 (14.8)	5.3 (10.8)	100 ± 6

Δ*mglBA*+*mglBA^G19A^*	MxH2445	NM	2.7 (11.8)	< 0.01

Δ*mglBA+mglBA^G21V^*	MxH2361	NM	2.8 (11.8)	0.01 ± 0.01

Δ*mglBA+mglBA^L22V^*	MxH2359	1.9 (20.6)	3.8 (12.0)	15 ± 4

Δ*mglBA+mglBA^K25A^*	MxH2430	NM	2.7 (10.5)	< 0.01

Δ*mglBA+mglBA^T26N^*	MxH2410	NM	1.4 (11.3)	< 0.01

Δ*mglBA+mglBA^D52A^*	MxH2408	NM	1.1 (10.3)	< 0.01

Δ*mglBA+mglBA^T54A^*	MxH2406	NM	2.0 (10.3)	< 0.01

Δ*mglBA+mglBA^T78A^*	MxH2247	0.7 (15.5)	3.0 (11.5)	15 ± 3

Δ*mglBA+mglBA^T78S^*	MxH2248	1.4 (21.8)	2.7 (7.8)	< 0.01

Δ*mglBA+mglBA^T78D^*	MxH2432	NM	NM	0.1 ± 0.0

Δ*mglBA+mglBA^P80A^*	MxH2357	NM	NM	20 ± 6

Δ*mglBA+mglBA^Q82A^*	MxH2320	NM	2.0 (8.0)	< 0.01

Δ*mglBA+mglBA^Q82R^*	MxH2319	NM	1.8 (10.3)	0.01 ± 0.0

Δ*mglBA+mglBA^L117/L120A^*	MxH2339	NM	1.4 (9.7)	< 0.01

Δ*mglBA+mglBA^L124K^*	MxH2279	3.6 (8.4)	5.0 (7.6)	< 0.01

Δ*mglBA+mglBA^N141A^*	MxH2338	NM	1.8 (9.8)	< 0.01

Δ*mglBA+mglBA^K142A^*	MxH2365	NM	2.5 (10.2)	< 0.01

Δ*mglBA+mglBA^D144A^*	MxH2367	NM	1.6 (10.6)	< 0.01

WT + *mglBA^+^*	MxH2375	2.1 (9.7)	8.9 (16.0)	40 ± 10.0

WT + *mglB^+^*	MxH2391	2.3 (20.0)	6.6 (15.0)	40 ± 10.0

WT*+mglBA^G19A^*	MxH2431	1.3 (20.8)	4.0 (19.7)	10 ± 0.6

WT*+mglBA^G21V^*	MxH2360	2.1 (18.2)	5.2 (15.3)	100 ± 12

WT*+mglBA^L22V^*	MxH2358	1.8 (15.3)	7.6 (17.5)	2 ± 1.5

WT*+mglBA^K25A^*	MxH2429	1.8 (21.3)	5.2 (13.6)	60 ± 15

WT*+mglBA^T26N^*	MxH2409	1.9 (21.0)	8.3 (12.5)	< 0.01

WT*+mglBA^D52A^*	MxH2407	NM	8.7 (12.4)	0.03 ± 0.01

WT*+mglBA^T54A^*	MxH2405	2.5 (16.2)	9.3 (14.4)	0.01 ± 0.0

WT*+mglBA^T78A^*	MxH2425	1.7 (25.0)	8.2 (13.4)	30 ± 6

WT*+mglBA^T78S^*	MxH2426	2.2 (21.4)	7.1 (15.5)	< 0.01

WT*+mglBA^T78D^*	MxH2428	NM	6.0 (12.6)	90 ± 5

WT*+mglBA^P80A^*	MxH2356	2.0 (23.6)	2.3 (18.3)	40 ± 6

WT*+mglBA^Q82A^*	MxH2404	1.6 (30.0)	7.5 (13.5)	< 0.01

WT*+mglBA^Q82R^*	MxH2368	2.6 (22.1)	10.0 (22.2)	100 ± 18

WT*+mglBA^L117/L120A^*	MxH2337	1.3 (15.6)	8.1 (18.4)	100 ± 18

WT*+mglBA^L124K^*	MxH2278	2.4 (15.1)	3.5 (15.4)	< 0.01

WT*+mglBA^N141A^*	MxH2336	1.7 (NR)	2.1 (17.2)	0.2 ± 0.2

WT*+mglBA^K142A^*	MxH2364	1.4 (21.3)	9.3 (17.6)	40 ± 6

WT*+mglBA^D144A^*	MxH2366	1.6 (22.5)	2.4 (11.5)	4 ± 1

Time-lapse microscopy was performed to determine the rates of gliding cells.

^a ^Gliding and reversal rates for cells using A-motility were measured on 1.5% CTPM agarose pads as described in Methods. NM = Cells were nonmotile. NR = no reversals observed.

^**b **^Gliding and reversal rates for cells using S-motility were measured in 0.5% methylcellulose plus 0.5× CTPM as described in Methods. NM = Cells were nonmotile. Gliding speeds are represented as the average and range of 25 cells from two independent assays.

^c^Sporulation rates are given as a percentage relative to the WT and the standard deviation if available. The ability of MglA mutants to complement the sporulation defects of the Δ*mglBA *mutant was performed as described in Methods. *mgl *alleles were introduced into the WT background to determine MglA mutants could interfere with the function of normal MglA during sporulation.

All three strains were examined for their ability to move as individual cells or in groups at the edge of a colony arising from a single cell. The colony edge morphology is illustrated in Figure 2C. A- and S-motility were restored (panel 3) to the Δ*mglBA *mutant when complemented with wild type *mglBA*, but addition of *mglBA *constructs with *mglA*-G19A, K25A and T26N failed to complement.

**Figure 3:**
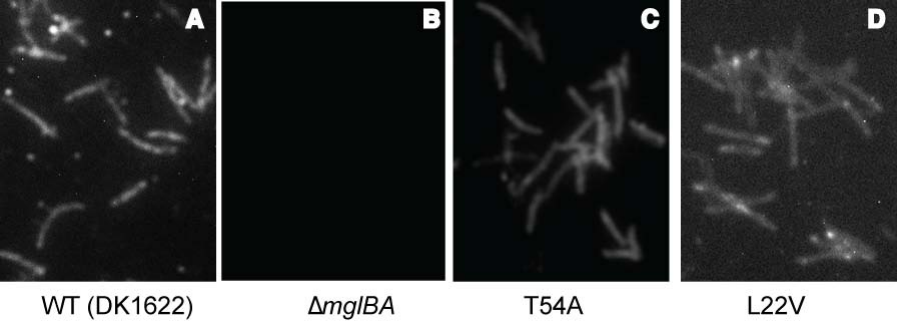
**Immunofluorescence of MglA demonstrates a change in localization in some MglA mutants**. Mutant *mglA-*containing strains were probed with an anti-MglA antibody after fixation as described in Methods. A. WT cells probed with anti-MglA antibody reveal a punctate distribution throughout the cell. B. *ΔmglBA *strain probed with anti-MglA antibody. No background fluorescence is observed. C. T54A cells probed with anti-MglA antibody. A diffuse fluorescence is observed with no punctate localization. D. L22V cells probed with anti-MglA antibody. Localization similar to that of the WT can be observed.

**Figure 4:**
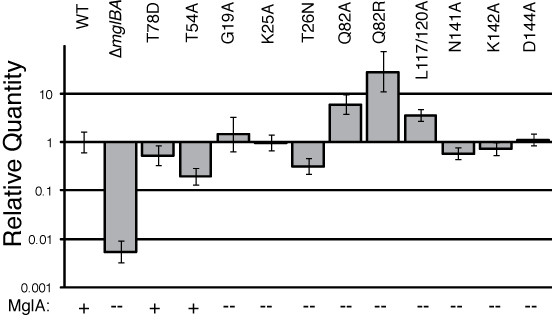
**Mutants that fail to produce MglA display increased transcript levels relative to the WT**. cDNA was produced from mRNA harvested from the WT, Δ*mglBA *mutant and complementing strains as described in Methods and analyzed by qRT-PCR (Applied Biosystems). Background fluorescence was subtracted using the no-template control (NTC), resulting in the data shown. The data (n = 6) shown are relative to the normalized WT (value = 1). In order, the bars represent the WT, DK6204, MxH2432 (T78D) and MxH2406 (T54A) as positive controls as mutants that make MglA in amounts detectable by Western blot, and the mutants G19A, K25A, T26N, Q82A, Q82R, L117/120A, N141A, K142A and D144A respectively. MglA: (+) = made MglA, (--) = did not make MglA.

To determine whether these mutants produced stable MglA, whole cell extracts were probed with α-MglA antibody. As shown in Figure 2D, MglA protein was not detected by Western blot analysis for any of the PM1 mutants relative to the loading control (sample Western with loading control is shown in Additional file 6: FigureS6 Western control). WT cells displayed a punctate distribution of MglA along the cells length as visible by immunofluoresence, as shown in Figure 3A. In contrast, the deletion parent mglBA did not produce MglA and showed no fluorescence relative to the background, Figure 3B. All PM1 mutations in conserved residues resembled the deletion parent as shown in Figure 3B. To investigate the possibility that lack of MglA was due to decreased transcription, we performed RT-PCR to obtain a quantitative measure of transcription from the *mgl *locus. Total mRNA was obtained from mid-log phase *M. xanthus *and isolated as described in Methods. The WT, Δ*mglBA*, two mutants that produce MglA without restoration of motility, and all mutants that failed to produce detectable amounts of MglA were assayed (Figure 4). The WT transcript level was normalized to one and all others are shown relative to the WT. In all cases that we examined, the inability to detect MglA on a Western blot was not due to a defect in transcription. In fact, both mutants assayed that made MglA showed a slight decrease in transcript level, while several mutants that failed to accumulate protein showed an increase in MglA transcript relative to the wild-type. In particular, both changes at codon 82 increased the amount of *mgl *transcript 10-fold. Upon *in silico *comparison of the predicted secondary structures of the *mgl *transcript from WT and Q82R (or Q82A), we found that the single base substitution significantly alters the topology of the structure predicted to have the lowest free energy, as displayed in Additional file 7: FigureS7 RNA. Hence, the Q82 mutations may prevent transcript degradation, which could account for the elevated levels of *mgl *mRNA detected in these mutants. It is conceivable that changes in the RNA structure might also affect translation, thus contributing to the absence of accumulated protein in these mutants.

### Mutants with altered G2 motif fail to complement the deletion parent

The NKxD residues are located close to the guanine base of the GTP molecule presented in the model of MglA (previously shown in Figure 1), suggesting an interaction with GTP similar to that described for Ha-Ras and other structural models [13]. The side chains of residues Asn116, Lys117 and Asp119 in Ha-Ras have been shown to exist within hydrogen-bonding distance of the base of GTP and are predicted to provide specificity for this purine over adenine [13]. Mutations that affect Asn116 and Asp119 in Ha-Ras result in an increased nucleotide dissociation rate *in vitro *[34,35].

**Figure 5:**
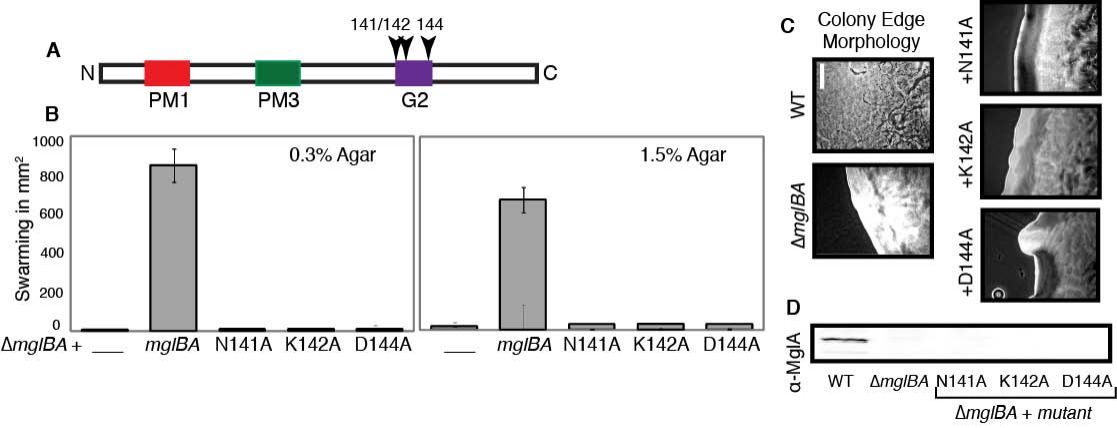
**G2 mutations fail to complement the motility defect of Δ*mglBA***. MglA alleles with mutations in residues Asn141, Lys142 and Asp144, which are predicted to interact with the guanine base of GTP fail to complement the deletion phenotype. Mutations shown in this panel are from the G2 region: MxH2338 (N141A), MxH2365 (K142A) and MxH2367 (D144A). The first two bars represent the Δ*mglBA *parent and control respectively. See Figure 2 legend.

Alanine subsitutions were constructed for each of the conserved residues in the corresponding NKxD motif of MglA from residues 141 to 144 to determine if altering the predicted guanine binding pocket would affect gliding (Figure 5A). Plasmids carrying these mutations were introduced into the Δ*mglBA *mutant and their phenotypes characterized as described above. Mutants N141A, K142A and D144A each produced colonies with smooth, even edges characteristic of a nonmotile colony (Figure 5C). As shown in Figure 5B, swarming of strains with N141A, K142A, and D144A alleles was <5% of the control on 1.5% agar and <2% of the control on 0.3% agar. No individual cell movement was seen by videomicroscopy on agarose and the oscillating movement of N141A, K142A, D144A mutants in MC was consistent with the behavior observed in the Δ*mglBA *deletion parent.

Strains with mutations in G2 failed to produce sufficient mutant MglA to be detected by Western blot as shown in Figure 5D. This result suggested that G2 residues may be critical for the stability of MglA, or that failure to accumulate MglA may be a result of a decrease in transcriptional activation from the *mgl *locus. Additionally, no mutant MglA was detected by immunofluorescence. All strains resembled the deletion parent, as shown previously in Figure 3B. As with the PM1 mutants above, we examined the G2 mutants for their *mglA *transcript levels. As shown in Figure 4, we confirmed that a loss of transcription activation probably does not account for the lack of MglA protein since *mgl *mRNA is found in comparable amounts to the WT. The inability to properly coordinate hydrogen bonds with the nucleotide may be responsible for our failure to detect MglA in the complementation strains as the protein may be unstable or misfolded without bound nucleotide.

### Mutations that correspond to activating mutations in certain monomeric GTPases affect the function of MglA

Well-characterized activating mutations (G12V, G13V, Q61A/L/R) in Ras-like GTPases are predicted to reduce the rate of GTP hydrolysis *in vivo *[13,30] and are GAP insensitive [36]. Residues in MglA that correspond to known activating (single or double) mutations at amino acids G12, G13, A59 and Q61 of Ha-Ras were engineered to make G21V, L22V, P80A, and Q82A (and 82R) changes, respectively, in *mglA*.

**Figure 6:**
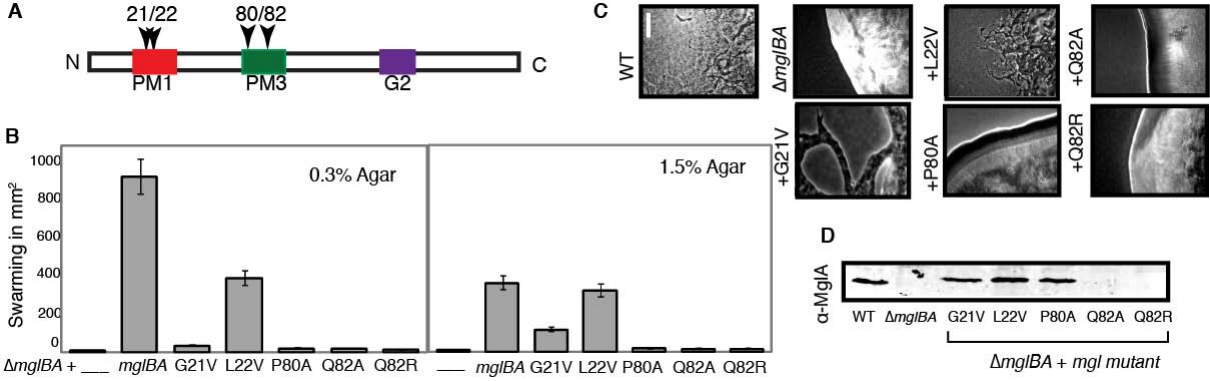
**Mutants with activating mutations display defects in one or both motility systems**. MglA alleles which were made to resemble activating mutations in Ras displayed decreased or absent motility in a complementing strain. Mutations shown in this figure include MxH2361 (G21V), MxH2359 (L22V), MxH2357 (P80A), MxH2320 (Q82A) and MxH2319 (Q82R). See Figure 2 legend.

Motile flares at the colony edges of MglAG21V and L22V strains showed that motility was restored partially (Figure 6C). Both mutants could swarm on 1.5% agar: swarms were 32% and 89% the level of the control for G21V and L22V, respectively as shown in Figure 6B. Both strains swarmed poorly on 0.3% agar, 3% and 37% that of the control for G21V and L22V, respectively, which suggests that both mutations exert stronger effects on S-motility than on A-motility.

Cells containing MglAG21V could neither move individually on a 1.5% agarose surface nor in 0.5% MC (videomicroscopy, Table 1), although stable MglA was produced and some flares were observed at the colony edge (third panel, Figure 6C). In contrast, videomicroscopy showed that the L22V mutant glided well on agarose (90% of the control) and showed speeds in methylcellulose of 71% of the control (Table 1). Reversals occurred less frequently in the L22V mutant (1 in 20.6 min, compared to 1 in 14.8 min for the control) in both agarose and in MC (1 in 12.0 min, compared with 1 in 10.8 min for control). Although these results would seem to contradict the swarming assay, we observed a density-dependent effect on motility in the microscopic assays. When cells were in contact, both G21V and L22V speeds increased and more closely correlated with their success in swarming assays.

The proline in PM3, P80, is conserved in proteins closely related to MglA as well as distant relatives LepA, Obg, Era and YihA. Many eukaryotic GTPases, such as those in the Rho, Ras and Rab families, contain an alanine in this position. The analogous residue A59 in Ha-Ras is involved in retaining GDP by preventing dissociation of the ligand by conformational change in Ha-Ras and mutation to threonine is considered an activating mutation [13]. To explore the possibility that substitution of the bulky proline in MglA might improve its function, P80 was changed to alanine. Although the P80A mutant improves the PM3 motif match with most eukaryotic, as well as many prokaryotic GTPases such as FtsY, YchF, and TrmE, this mutation completely abolished MglA function *in vivo *despite the fact that stable MglA protein was made (Figure 6D). The P80A mutant was mot^- ^and dev^-^.

MglAQ82 mutants were expected to reduce the rate of GTP hydrolysis based on the effect of the analogous change in Ras (Q61). Initially Q82R was made to mimic known Ras mutants but this mutant allele failed to produce detectable MglA (Figure 6D) and the strain was nonmotile. Subsequently, Q82A was made to offset concerns that the charged arginine in this position inhibited folding of MglA. Unfortunately, the Q82A mutant likewise did not produce MglA and the phenotype was indistinguishable from the parent. Zhang *et al*. reported stable MglAQ82L expressed from the *att *site, however our constructed mutants (which integrated at the chromosomal site) failed to accumulate stable MglA protein [18]. Time-lapse microscopy failed to detect any movement on 1.5% agarose for either strain; motility in MC was nearly identical with the parent. Loss of transcript did not appear to account for the problem because, as shown in Figure 4, the levels of *mglA *transcript for both the Q82A and Q82R were found to be elevated. The apparent increase in mRNA level by qRT-PCR and, paradoxically, the decreased expression of MglA may be due to alterations in the predicted secondary structure of *mgl *RNA resulting from codon 82 modifications.

All activating mutation strains were assayed for their localization. We did not detect MglA in the Q82 mutants, consistent with the Western blot showed in Figure 6D. In the G21V and L22V, we observed localization as previously seen in Figure 3D, which depicts the L22V localization pattern. The localization pattern for P80A was indistinguishable from the WT (WT shown in Figure 3A).

### Mutations that are predicted to affect surface residues alter or decrease MglA function and may affect protein-protein interactions

**Figure 7:**
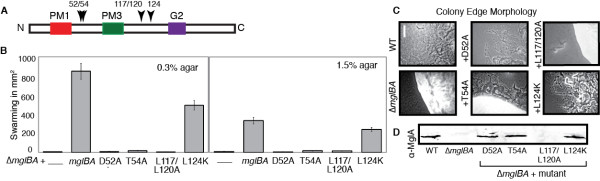
**Mutations predicted to alter surface residues abolish function of MglA**. Residues predicted to exist on the surface of MglA either failed to complement the deletion phenotype or partially restored the activity of both motility systems. Strains in this panel include MxH2408 (D52A), MxH2406 (T54A), MxH2339 (L117/120A) and MxH2279 (L124K). See Figure 2 legend.

Based on the three-dimensional model of MglA (Figure 1), we predicted that residues Asp52, Thr54, Leu117, Leu120 and Leu124 might be surface exposed. Asp52 and Thr54 lie within a region that corresponds with a GAP (GTPase Activating Protein) effector-binding region of eukaryotic GTPases [36]. Leu117, Leu120 and Leu124 are three of the leucines that comprise a short stretch between Leu117 and Leu145 that resembles a leucine repeat (Lx_6_L) [37] that are likely to reside on a single face of an α-helix. These hydrophobic residues and their neighbors would either be buried in the interior of the protein or would indicate a potential binding site for an interacting protein with a similar hydrophobic face. The residues in this leucine-rich repeat (LRR) were indicated in orange in Figure 1 and are highlighted in Figure 7A. The role of each of these residues in gliding and development was investigated.

Residues D52 and T54 were found to be critical for the function of MglA. Both mutants produced stable MglA protein that had significantly reduced function. Although some gliding flares (including isolated cells) were apparent at the colony edge of each mutant strain (Figure 7C), swarming was abolished (Figure 7B). Both strains displayed oscillating motions in methylcellulose, showing a far increased frequency of reversals indicative of the parental phenotype (~1 per 10 min), and it showed motility in microscopic assays similar to the parent strain. Because of this frequency, we believe that progressive movement from the central spot is less efficient, i.e., net movement as measured by the swarming assay is decreased. Because both D52A and T54A mutants behaved like the deletion parent, yet make MglA protein, we investigated whether the localization pattern was different in these mutants. Indeed, both D52A and T54A produced a diffuse staining pattern with anti-MglA, which suggests that these mutations, which lie on a predicted recruitment interface of MglA, profoundly affect the ability of MglA to interact with a partner. A representative T54A IF is shown in Figure 3C. The diffuse pattern was seen for only one other mutant, MglAD52A. In contrast, other mutants that make MglA, such as L22V, exhibited a pattern of localization that was similar to the WT (as previously shown in Figure 3D).

Candidate surface-exposed leucine residues of MglA were changed in an attempt to identify potential protein binding sites. While single mutations at L117 or L120 had a mild effect on the function of MglA (single mutants displayed near-WT motility; data not shown), the L117A/120A double mutant strain failed to produce detectable MglA protein, despite the fact that transcript was made (as previously shown in Figure 4). Consistent with all other mutants that fail to make MglA protein, the L117A/L120A mutant was nonmotile (Figure 7, Table 1). By contrast, colonies of the L124K mutant, which made MglA protein, had WT-like flares and mutant cells swarmed on 1.5% agar (70% of control) and 0.3% agar (50% of control). In microscopic assays, the L124K mutant demonstrated robust gliding on 1.5% agarose (Table 1), exceeding the control by 2-fold. Movement in MC was 94% of the control. The reversal frequency was elevated in this mutant - cells reversed every 8.4 min on agarose, about half that of the control (1 in 14.8 min) and every 7.6 min in MC compared to 1 in 10.8 min for the control. This might account for the decrease in swarming, particularly on 0.3% agar.

### Amino acid residue Thr78 is conserved among a group of MglA-like proteins and is essential for motility

The PM3 region of all Ras superfamily GTPases characterized to date have the consensus sequence DxxG. In contrast, the corresponding region of MglA has the sequence TxxG. This distinguishing feature is not an anomaly since homologs of MglA found in other bacteria all contain the TxxG sequence (Table 2) [38,39] and may define a new subfamily of small GTPases.

**Table 2:** Diverse prokaryotes encode an MglA-like protein.

Organism	Accession	Amino acids	MglB partner?^*a*^	Identity^*b*^	Positives^*b*^
**Group I: MglA proteins**					

*Myxococcus xanthus*	AAA25389	195	Yes	100%	100%

*Anaeromyxobacter dehalogenans *2CP-C	EAL78512	195	Yes	171/195 (87%)	186/195 (95%)

*Geobacter sulfurreducens*	NP_951161.1	195	Yes	160/194 (82%)	179/194 (92%)

*Geobacter metallireducens*	ZP_00080325.1	195	Yes	156/194 (80%)	178/194 (91%)

*Sorangium cellulosum *So ce26	AAR25888.1	196	Yes	160/193 (82%)	175/193 (90%)

*Bdellovibrio bacteriovorus*	NP_970444.1	197	No	126/194 (64%)	161/194 (92%)

*Deinococcus radiodurans*	NP_294577.1	196	Yes	119/194 (61%)	156/194 (80%)

*Thermus thermophilus*	AP008226.1	196	Yes	121/195 (62%)	153/192 (78%)

*Chloroflexus aurantiacus*	YP_001635661.1	195	Yes	105/195 (53%)	142/195 (72%)

*Desulfotalea psychrophila *LSv54	YP_066512.1	201	Yes	91/202 (45%)	127/202 (62%)

*Aquifex aeolicus *VF5	NP_214074.1	190	No	81/186 (43%)	115/186 (61%)

**Group II: MglA2 proteins**					

*Fibrobacter succinogenes*	CP001792.1	313	No	119/192 (58%)	149/192 (78%)

*Myxococcus xanthus*	AAL56599.1	281	No	81/182 (44%)	120/182 (65%)

*Geobacter metallireducens*	ZP_00080378.1	225	No	82/180 (45%)	112/180 (62%)

*Geobacter sulfurreducens*	NP_952979.1	291	No	76/192 (39%)	113/192 (58%)

**Eukaryotic GTPases related to MglA proteins**					

*Ustilago maydis*	EAK87233.1	189	No	43/151 (28%)	72/151 (47%)

*Saccharomyces cerevisiae Sar1p*	NP_015106.1	190	No	46/157 (29%)	69/157 (43%)

*Dictyostelium discoideum *AX4 ADP-ribosylation-like protein 8	XP_639087.1	185	No	43/141 (30%)	70/141 (49%)

^a ^MglB partner is denoted as an open reading frame immediately upstream from MglA with an identifiable Roadblock/LC7 motif. ^b^Values for identity and positives (similarity) are relative to the 195 amino acid protein MglA from *Myxococcus xanthus*. BLAST analysis was performed as described [63]. Identity and positives show the number of identical (positive) residues as a fraction of the total number of residues used for alignment. This fraction is given beneath as a percentage.

**Figure 8:**
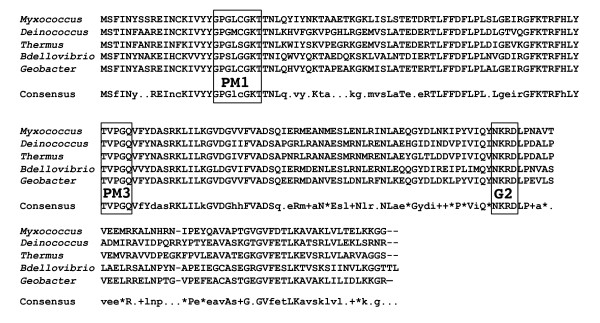
**MglA represents a new family of monomeric GTPases in prokaryotes**. Shown is the alignment of the predicted sequences of MglA from *M. xanthus *with *Deinococccus radiodurans, Thermus thermophilus, Bdellovibrio bacteriovorus, and Geobacter metallireducens*. Conserved sequence elements (PM1, PM3 and G2) for GTP binding are boxed. Consensus: Upper case letter = conserved in all five proteins listed; lower case letter = conserved in at least 3 of 5 proteins; * = conservative substitution; + = semi-conservative substitution; . = no conservation.

The MglA-like proteins fall into two groups based on their sizes. Group 1 proteins range in size from 190 to 197 amino acids, similar to Ras (189 amino acids). Group 2 proteins range in size from 225 to 327 amino acids. Homologs in this group have additional C-terminal domain of unknown function. A comparison of identity and similarity between *M. xanthus *MglA and its group 1 and 2 homologs, including those from *Geobacter sulfurreducens, Bdellovibrio bacteriovorus, Thermus thermophilus*, and *Chloroflexus aurantiacus*, is shown in Table 2. An alignment between *M. xanthus *MglA and its group 1 homologs, including those from *G. metallireducens, B. bacteriovorus, T. thermophilus*, and *Deinococcus radiodurans*, is shown in Figure 8.

**Figure 9:**
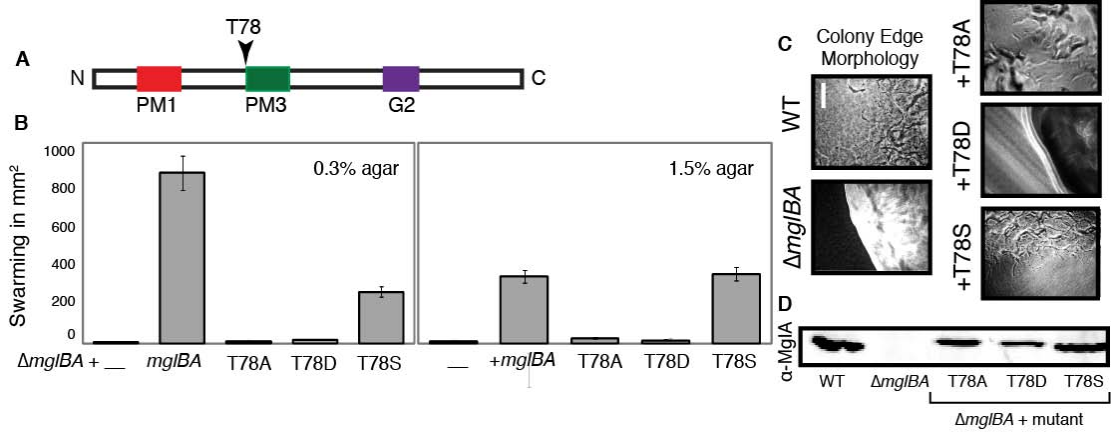
**Mutations in T78 demonstrate the requirement of a novel PM3 substitution**. This panel shows the phenotypes of strains MxH2247 (T78A), MxH2432 (T78D) and MxH2248 (T78S). See Figure 2 legend.

As the substitution of a polar threonine residue for an acidic aspartate residue in the PM3 region (highlighted in Figure 9A) is unusual among the small GTPases in the Ras superfamily, we investigated the role of this residue in the function of MglA. Surprisingly, the MglAT78D modification, which perfects the overall consensus with all other GTPases (outside of the MglA group), abolished the activity of MglA, even though MglA protein was produced (Figure 9D) and yielded a localization pattern similar to the WT (as previously shown in Figure 3A). The T78D mutant had an even, smooth border (Figure 9C) and was unable to swarm (Figure 9B). Additionally, motility on 1.5% agarose and in MC was completely abolished (Table 1).

Other substitutions at Thr78 had less severe effects. The motility defect of a Δ*mglBA *strain was complemented only poorly by the *mglA*T78A allele, which also makes MglA protein (Figure 9D). Although small flares, suggestive of S-motility, were present at the edges of colonies formed by strain MxH2247 (Figure 9C), the swarming rates were very low (Figure 9B). Isolated cells characteristic of A-motility were not seen at the edges of MxH2247 colonies although some movement was observed by videomicroscopy on 1.5% agarose (0.7 ± 1.1 μm/min). Gliding in MC (3.0 ± 1.4 μm/min) was only marginally better than the Δ*mgl *parent.

A conservative threonine to serine substitution yielded stable, functional MglA. As shown in Figure 8C, the edge morphology of MxH2248 (MglAT78S) was indistinguishable from the WT. Swarming of the T78S mutant was 100% of the control strain on a 1.5% agar but only 26% of the control on 0.3% agar suggesting that S-motility is impaired specifically in this mutant (Figure 9B). Consistent with this, videomicroscopy showed that the T78S mutant restored gliding speeds to 66% of the control on agarose (A-motility) but gliding rates on MC were only 56% of the control.

### Some *mglA *mutants impart a dominant negative phenotype

Mutations in *mglA *that alter residues critical for protein interaction might have a dominant effect on motility and can be useful tools to identify protein partners and suppressors. To identify such residues and determine the phenotype of mutant forms of MglA in the presence of WT MglA, we constructed merodiploid strains. Mutant alleles of *mglA *with normal *mglB *and the *mgl *regulatory region were integrated at the chromosomal site of DK1622 (*mglB^+^A^+^)*, resulting in two tandem copies of *mglB *and *mglA *each expressed from the *mgl *promoter. Two additional controls were included in these assays to examine the effect of multiple copies of *mglB *and *mglA *on motility. One strain (MxH2375) contained two WT copies of *mglBA *and one strain (MxH2391) contained an additional copy of *mglB*, to simulate the effects of a merodiploid that carries an allele of *mglA *that fails to produce stable MglA protein, but produces extra MglB.

**Figure 10:**
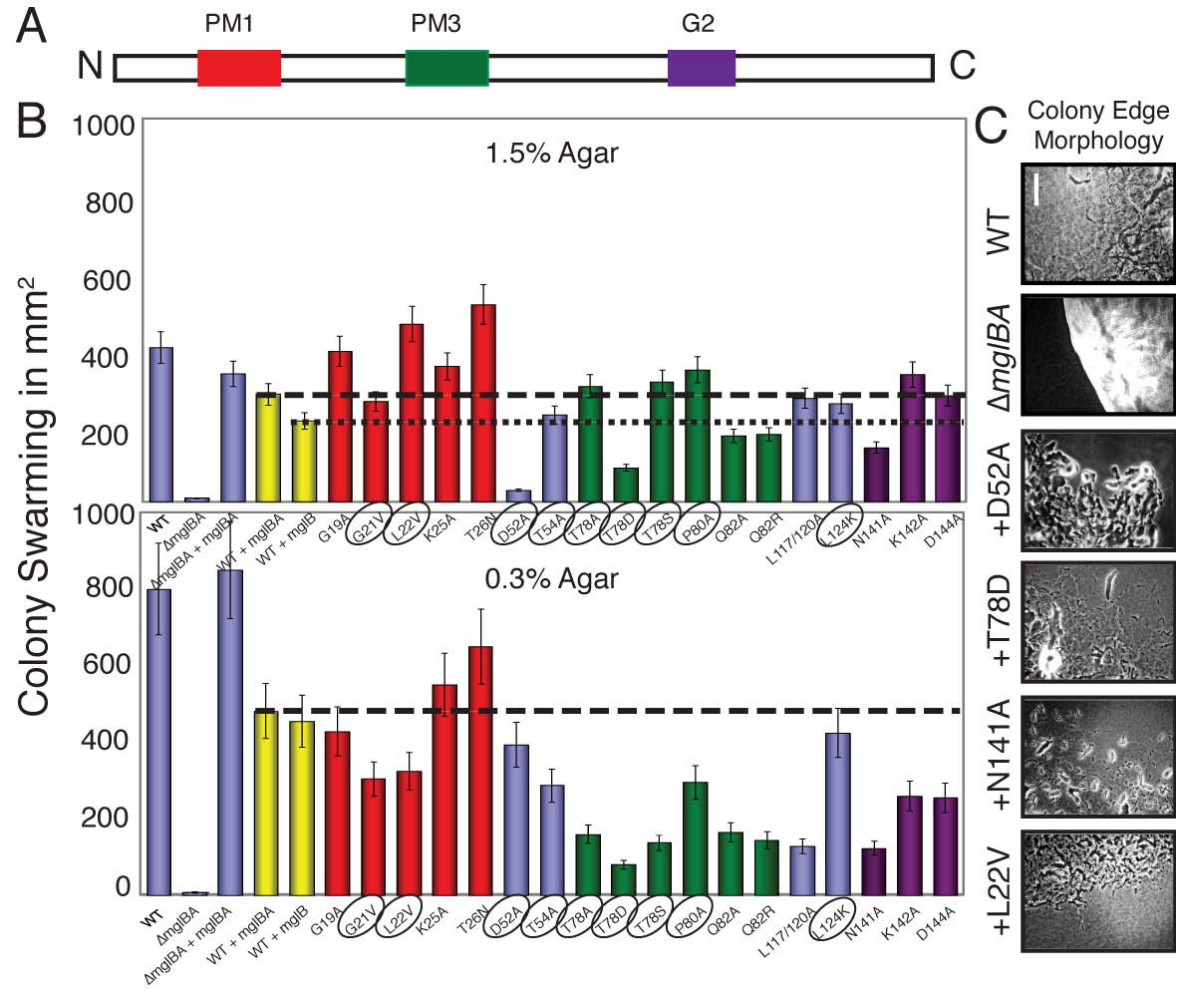
**Some MglA point mutations give a dominant-negative phenotype**. Addition of a second copy of the *mgl *locus depressed the motility phenotypes of merodiploids. A: Linear model of MglA. B: Swarming on 1.5% CTPM agar (top graph) and 0.3% CTPM agar (bottom graph). Bars are colored with respect to location within a conserved motif (red for PM1, green for PM3 and purple for G2), matching the colors used in Figure 10A. Yellow bars represent the *mgl *merodiploids MxH2375 (WT+*mglBA*) and MxH2391 (WT+*mglB*) respectively. The dashed lines provide comparison to merodiploid control, while the dotted line in the upper panel provides comparison to MxH2391. Strains which make detectable mutant MglA in complementing strains are circled. C. Colony edge morphology of selected merodiploids relative to WT and merodiploid controls. Pictures were obtained from isolated colonies on 1.5% CTPM agar at 100× magnification. Bar = 25 μm.

As shown in Figure 10, deletion of *mglBA *abolishes swarming that is restored to near WT levels upon addition of *mglBA*. The presence of an extra copy of *mglBA *or *mglB *introduced to the wild-type parent (MxH2375 and MxH2391, respectively) decreased swarming by 13% and 40%, respectively, on 1.5% agar and by 47% and 50% respectively on 0.3% agar relative to WT. While the gliding speed on 1.5% agarose was not severely affected by the addition of a second copy of the full *mgl *locus (81% of WT), cells reversed twice as often in the full *mgl *merodiploid on 1.5% agarose (1 in 9.7 min), compared to the WT (1 in 20.7 min). In contrast, addition of a second copy of *mglBA *caused the rate of gliding in 0.5% MC to double (185% of WT speed), yet the reversal frequency in MC was unchanged. Similarly, the addition of a second copy of *mglB *only had minimal impact on gliding on agarose (88% of WT) and modestly improved gliding speed in MC (138%). Reversal frequencies were unchanged. The mechanism by which additional MglB and MglA affect motility is still being explored but if MglB from *M. xanthus *has GAP activity as reported for the related MglB from *Thermus *and MglB from *M. xanthus *[18,19], extra copies of MglB might deplete the amount (or duration) of active (GTP bound) MglA in the cell. Our results suggest that this affects swarming without significantly affecting the motor rates.

For the purposes of this investigation, the merodiploid strains containing mutant alleles of *mglA *are compared to MxH2375, the merodiploid containing two full copies of the *mgl *locus, referred to hereafter as the merodiploid control. The first two bars displayed in Figure 10 show swarm data for the WT and deletion parent, followed by the complement control, the WT merodiploid MxH2375 and the *mglB *merodiploid MxH2391 respectively. Merodiploid controls are shown in yellow. The remaining colors are grouped according to recognized monomeric GTPase motifs. Among the merodiploids carrying mutant alleles, five phenotypes were observed based on colony swarming and motility rates: little or no change relative to the control, improved swarming, reduced A-motility (1.5% agar), reduced S-motility (0.3% agar) and reduced A and S-motility. In the analysis, we took into account that changes in swarming might be attributed to additional MglB for the nine constructs for which the mutated allele of *mglA *fails to produce stable protein. These nine strains produced normal MglB and MglA, plus additional MglB. The remaining strains produced additional MglB and mutant MglA.

The swarming capability on 1.5% agar for strains that made mutant MglA protein was compared with the WT carrying extra wild-type *mglBA *(Figure 10A, dashed line). MglAD52A and MglAT78D were dominant to MglA, inhibiting A-motility by >80%. With regard to D52A, the result hints that the putative recruitment interface, where D52A maps, is important for MglA interactions with an A-motility protein, such as AglZ. The fact that MglAD52A interferes with normal MglA function, perhaps through sequestration by a putative partner, also explains why MglAD52A in single copy abolishes both A and S motility. The behavior of the T78D mutant, whether it is with or without WT MglA, suggests that it also might interfere with MglA's partners.

One mutant, MglAL22V, had a stimulatory effect. For other MglA-producing strains, swarming was comparable to the control. As described above, swarming on 1.5% agar was reduced in strains with a second copy of *mglB *(Figure 10A, dotted line). With this in mind, we compared swarming of strains that harbor unstable forms of MglA. The phenotypes of five mutants were more severe than the control. Strains carrying Q82A/R and N141A inhibited swarming slightly while MglAG19A and T26N stimulated swarming. These differences might result from modest changes in transcription of *mglBA *or to transient production of mutant MglA.

Surprisingly, swarming on 0.3% agar was inhibited in a majority of the merodiploid constructs, which suggests that anything that perturbs MglA has a more profound impact on S-motility. This effect is not due to the extra copy of *mglB *because there was no significant difference between MxH2375 (WT + *mglBA*) and MxH2391 (WT + *mglB*) (Figure 10B and Table 1). MglAT78D, which was dominant to MglA for A-motility (Figure 10A and Table 1), was also dominant with regard to swarming on 0.3% agar, although cells showed near normal activity or an increase in velocity in MC by the microscopic motility assay (Table 1). Although there was no strict correlation between genetic dominance and the production of stable mutant MglA or transcript, we noticed that mutations that had a pronounced effect on gliding were clustered in the second half of the protein. In these mutants, a sufficient amount of the N-terminus of MglA might be made and folded to produce the inhibitory effect seen in these mutants. If this simple interpretation is correct, it would suggest that the N-terminal region of MglA regulates S-motility directly or indirectly.

### Alteration in MglA function produces aberrant fruiting body morphology

**Figure 11:**
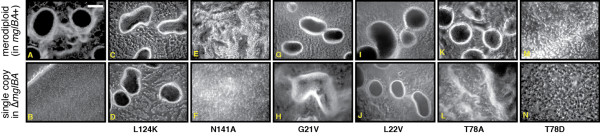
**MglA mutations abolish or alter fruiting body formation**. Fruiting body formation of mglA mutants was compared with the WT strain on TPM starvation medium containing 1.5% agar as described in Methods. a) Wild type DK1622(*mgl*+). b) DK6204 (*mgl*-) c. MxH2278 (*mglA + mglA*-L124K merodiploid). d). MxH2279 (*mglA- + mglA*-L124K). e). MxH2336 (*mglA + mglA*-N141A merodiploid). f). MxH2338 (*mglA- + mglA*-N141A). g). MxH2360 (*mglA + mglA*-G21V merodiploid). h). MxH2361 (*mglA- + mglA*-G21V). i). MxH2358 (*mglA + mglA*-L22V merodiploid). j). MxH2359 (*mglA- + mglA*-L22V). k). MxH2425 (*mglA + mglA*-T78A merodiploid). l). MxH2247 (*mglA- + mglA*-T78A). m). MxH2428 (*mglA + mglA*-T78D merodiploid). n). MxH2432 (*mglA- + mglA*-T78D). Photographs were taken with a Nikon FXA microscope at 100× magnification. Bar = 50 μm.

Previous studies have shown that MglA is required for proper fruiting body formation and sporogenesis when cells are starved for nutrients [23]. Each of the mutant strains was assayed for their ability to aggregate and form fruiting bodies on starvation medium. After 5 days, developing samples were heated and the number of heat-resistant spores was quantified. As shown in Figure 11, fruiting bodies containing refractile spores were present in the WT strain (A) but not in the Δ*mglBA *mutant (B). The deletion strain had less than 0.01% of the WT number of spores whereas the complementing control produced the WT number of spores. Representative microphotographs of developing samples are show in Figure 11. Sporulation efficiency is presented in Table 1.

Mutants that failed to produce detectable MglA (nine total) were unable to develop fruits or spores and resembled the Δ*mglBA *parent (Figure 11B). A representative of this group is shown in Figure 11F (N141A mutant). Of the mutants that made MglA protein (nine total), two mutants, L124K (Figure 11D) and L22V (Figure 11J), produced dark fruit that resembled the control, but were slightly smaller in size. All other MglA-producing strains produced only weak mounds (G21V, Figure 11H) or failed to produce mounds at all (N141A, T78A, T78D, Figure 11F, L, and 11N). The developmental defect associated with T78A was in sharp contrast with the T78S phenotype, which produced mature dark fruit identical to the control (data not shown). Sporulation was affected in all of the *mglA *mutants (Table 1). One possible explanation for why most *mglA *mutants failed to produce spores may be due to the fact that there was a decreased frequency of phase variation observed in certain *mglA *mutants. These remained phase-stable in a yellow variant, while strains that did form spores seemed capable of more regular variation between tan and yellow variants (data not shown). Additionally, the stability of wild-type MglA was examined during a period of 24 hours after the onset of starvation. There is a marked decrease in the amount of MglA detectable by Western blot by sixteen hours as shown in Additional file 8: FigureS8development. Previous experiments have shown a depletion of GTP during starvation related to the formation of a messenger ppGpp(p) in *M. xanthus *that may explain this observed degradation of MglA. If GTP is important for the stability of MglA, it is likely that any depletion or sequestration would also lead to a degradation of the protein.

A subset of MglA mutants interfered with the function of normal MglA to form fruiting bodies and heat-resistant spores. The presence of three MglA mutants, L124K, G21V and T78A (Figure 11C, G, K) resulted in fruiting bodies that were smaller than the control while two mutants, N141A and T78D (Figure 11E,11M) abolished the ability of normal MglA to produce fruit. The ability to form fruiting bodies did not necessarily correlate with ability to form spores in the merodiploid strains. Half of the merodiploids showed near-normal spore efficiency (30-100% of WT) and a few mutants produced a reduced complement of heat-resistant spores (1- 10%) (Table 1). Germination of heat-treated spores was reduced over 3-fold in six merodiploids containing the mutations T26N, D52A, T54A, T78D, Q82A, and L124K. We find this result puzzling because four of these mutants make stable mutant MglA protein but the remaining two do not make MglA on vegetative plate medium. Moreover, fruiting body formation was adversely affected in only two of the mutants in this group. Work is underway to determine how these residues affect the function of role MglA during sporogenesis.

## Conclusions

MglA is a small GTPase that is required for gliding motility and starvation-induced fruiting body development, but not growth, of *M. xanthus*. Previous work showed that nearly all known *mglA *mutants failed to make detectable protein [22,23] which has complicated the genetic structure-function analysis of MglA. To determine if forms of MglA could be identified that specifically affected A-motility, S-motility, or both, we used site-directed mutagenesis to generate a new collection of mutants.

**Figure 12:**
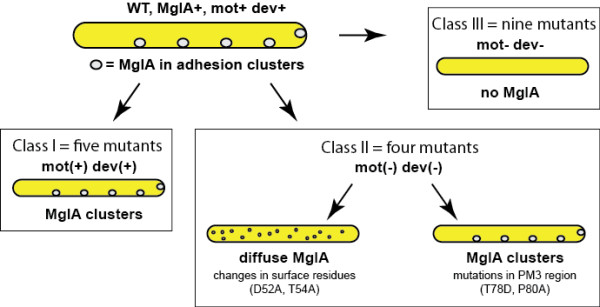
**Summary of mutations in MglA and their corresponding phenotypes with regard to *M. xanthus *motility**. Sixteen residues on WT MglA were targeted to make 18 point mutants. Nine mutants made MglA protein and were divided into groups based on phenotype and distribution of MglA (mot- (nonmotile), swm- (do not swarm), dev- (do not develop) and spo- (do not sporulate). Mutants that showed near WT phenotypes were scored as mot+ dev+. Those that showed only partial restoration of a characteristic were scored as (+). Those showed restoration of motility are called class I mutants, those that did not show a full restoration of motility are class II mutants. A subset of class II mutants which include the surface mutants D52A and T54A fail to localize correctly as identified using immunofluorescence microscopy. The remaining class II mutants localize correctly, but do not restore motility. The remaining nine point mutants failed to accumulate detectable amounts of MglA and are classified as class III mutants, which are mot- and dev-. Localization patterns are shown for each motility phenotype and mutant class.

Mutants fell into three general classes based on the ability of plasmids bearing p*mgl, mglB *and mutant *mglA *alleles to complement the defects of the Δ*mglBA *mutant. Class I mutants (five strains) made MglA protein and were able to swarm on surfaces and develop to some extent. Class II mutants (four strains) made MglA protein but did not swarm on surfaces or develop. Class III mutants (nine strains) failed to produce MglA protein and were unable to glide on surfaces, swarm, or develop fruiting bodies. For clarification, a flowchart is provided as Figure 12.

Mutations at one position, Thr78, yielded mutants in classes I and II. Thr78 is conserved in the MglA homologs found in bacteria, but it represents a significant departure from the consensus found in all other prokaryotic and eukaryotic GTPases, which use an aspartate in this position. MglA could tolerate serine in this position, but alanine and asparate abolished activity. Thr78 may represent a target for modification in MglA or may be essential for the interaction between MglA and critical effector proteins. Mutations in Ras that correspond with this region of the MglA protein are known to render Ras insensitive to GAP proteins [36,40], thereby affecting the rate of GTP hydrolysis *in vivo *by interaction with a critical surface feature of Ras-GAP known as the "arginine finger" [41]. Thus, the change of Thr78 to Asp may affect the ability of MglA to interact with other proteins *in vivo*. Consistent with this idea, we found that T78D was dominant to WT MglA for motility and development. These results show that threonine is critical for activity and suggest that MglA and its homologs represent a novel subfamily of GTPases.

Activating mutations are predicted to shift the balance to favor more of the GTP-bound (on) state of the GTPase. While it is not possible to make a global generalization, since some of the activating mutants failed to make protein, mutants with G21V and L22V made protein and were partially motile. The phenotype of the L22V mutant was less severe than that of the G21V mutant, a result that is consistent with the phenotypes reported for eukaryotic GTPases [42].

G21V was a mutation based on G12V of Ras, which decreases the rate of hydrolysis, a fact confirmed in a bacterial MglA from *Thermus thermophilus*. k_cat _for a G21V mutant was 7 times lower than that of WT MglA [19]. They also reported individual movement on buffered 1.0% agar slabs. In contrast, we saw predominantly social motility in our microscopic assays, with few individually moving cells (<5%). As previously discussed, the differences in nutritional conditions as well as agar content may dictate which motility system is active. However, Leonardy *et al*. did not investigate the effect on motility under conditions where social motility was favored.

Additionally, Leonardy *et al*. constructed a T26/27N double mutant while we constructed a single T26N mutation. Their mutant showed increased stability relative to our T26N mutant but was completely non-motile under all conditions they assayed. Their *Thermus *MglA carrying this mutation showed a further decrease in hydrolysis relative to both WT and G21V activating mutation, but also showed a substantial decrease in affinity for mantGTP and the non-hydrolyzable analog mantGPPNHP [19].

A subset of mutations predicted to disrupt surface residues yielded strains with potentially informative phenotypes. The substitution at Leu124, which may be part of a LRR, might alter the interactions with an effector protein. One candidate is AglZ, a protein known to interact with MglA [43], which contains heptad repeats that are characteristic of LRR-domain protein partners. Potential cycling of the MglA, AglZ, and FrzS triumvirate may yield clues to the regulation of A- and S-motility. Mauriello *et al*. confirmed the interaction of AglZ and MglA, as well as FrzS and MglA using tandem affinity purification [4]. If the L124K substitution altered the affinity of MglA for AglZ, this might perturb the interaction between AglZ and FrzS and might explain why the L124K mutant showed increased frequencies of cell reversal, however further investigation will be necessary to characterize the nature of this perturbation.

Two mutations in MglA altered the ability to localize correctly as observed by immunofluorescence. Both of the mutations which appeared to disrupt correct localization were predicted to be located on the surface of the protein, and on one face. One critical residue, D52, is analogous to the D33 residue in Ras, which has been shown to interact with a lysine in the protein NORE1A. NORE1A is a cytoskeletal protein that has been shown to be a suppressor of growth and oncogenic properties of active Ras [44]. It is possible that mutation of D52 in MglA has disrupted a similar protein interaction which would account for its lack of proper localization and function in a complementation background, and also the mutation's effects on the ability of *M. xanthus *to control reversal. We posit that the surface containing both D52 and T54 is responsible for proper recruitment of MglA to the cytoskeleton and that proper localization along the cytoskeleton is required for control of A-motility as well as regulating cell reversal.

The failure of class III mutants to make detectable MglA was surprising as similar sets of mutations in other monomeric GTPases have not been reported to affect protein stability. Introduction of polar residues in critical residues of Ha-Ras (N116K/Y) created a protein that was unable to bind GTP correctly, but did not alter stability [45]. Replacement with other large nonpolar or charged groups also altered GTP binding, but mutant proteins were stable *in vitro *[35]. This suggests that GTP binding itself has the potential to regulate the function of MglA in motility and development. Depletion of GTP by RelA as the cells respond to the starvation cue [46-48] might serve to shift motility patterns by reducing the amount of available MglA during development. The apparent disappearance of MglA during development would tend to suggest that a lack of GTP and the subsequent proteolysis of MglA may provide an internal timeline for proper development. Mutations that affect the ability of MglA to bind GTP may disrupt this process by allowing the premature degradation of MglA before spore maturation can occur. This observation represents a fundamental difference between MglA and other GTPases that may provide clues to the evolution of this group of protein.

Zhang *et al*. recently reported the phenotype of an MglAQ82L mutant, though no GTP hydrolysis rates were given [18]. This was another predicted activating mutation, similar to that of Q61L of Ras. It is possible that their mutant was stabilized by replacement with a leucine, similar to that seen in other mutants where the character of a mutation may stabilize the protein while affecting binding affinity. Our mutants at this location were actively transcribed, but appeared to be unstable, as no MglAQ82A/R was detectable by Western blot in three separate assays.

With regard to the merodiploid strains, which were constructed to look for dominance, we noted that perturbations in the balance of products from the *mgl *operon had a noticeable effect on motility. The presence of an extra copy of *mglB *inhibited the ability of merodiploid strains to swarm on 0.3% agar regardless of whether an extra copy of *mglA *was present. Therefore, balance of products from the *mgl *operon and other motility components may be critical for proper regulation of social motility in *M. xanthus*. The dominance screen yielded new tools for future studies. A predicted surface residue, D52, has potential for identifying protein partners for MglA because it was essential for gliding in the haploid and MglA-D52A abolished A-motility in the merodiploid. Similarly, the critical threonine at position 78 affected both A and S motility when MglA-T78D was paired with normal MglA.

While it is possible that overall dominant effects on S-motility are due to sequestration of gliding motor or regulatory components, research in other organisms has shown that the formation of a GTPase homodimer may be important for function. Dimerization has been observed to increase hydrolysis roughly twofold in *at*Toc33, a GTPase involved in protein import into chloroplasts [49]. Crystal structures show that Era and XAB1/MBD can each form dimers [50,51]. Although no crystal structure exists for MglA yet, it is possible that the dominant effects observed in our merodiploid mutant strains may be due to a decrease in the ability of MglA to function as a dimer in the regulation of motility and development.

Homologs of MglA found among the genomes of a diverse group of prokaryotes will likely provide clues to the evolution of this group of proteins. Based on the high degree of conservation among predicted MglA homologs, it is possible that these proteins share a common function. It is intriguing that all of the organisms identified to date that have homologs of *mglA *also carry structural genes for the assembly of Tfp. *Anaeromyxobacter dehalogens *[52], *Geobacter metallireducens *[53], and members of the related genera *Deinococcus *and *Thermus*, have genes encoding Tfp [54]. Similarly, some genes for Tfp determinants are found in the genome of the filamentous glider *Chloroflexus aurantiacus*, an anoxygenic, thermophilic photosynthetic bacterium [55]. Not all organisms that have Tfp have an *mglA *homolog, nor is it clear that all organisms encoding Tfp use these components for motility. For example, *Thermus thermophilus *uses Tfp machinery for DNA transfer [56]. Future studies might reveal a novel pathway by which these unusual GTPases regulate Tfp components in organisms from diverse habitats and in diverse functions.

## Methods

### Strains and media

Strains and plasmids are listed in Additional file 9: Table S1. *M. xanthus *strains were grown routinely in vegetative CTPM (1% Casitone, 10 mM Tris, 1 mM potassium phosphate, 5 mM MgSO_4_, final pH 7.6) medium at 32°. Unless otherwise noted, solid medium contained 1.5% agar. *E. coli *strains were grown in LB medium [57] at 37°, and were used for plasmid constructions and DNA purifications. When appropriate, media were supplemented with kanamycin sulfate (Kan; 40 μg/ml).

### Construction of plasmids with mutations in *mglA *and recombination of mutant alleles of *mglA *into the *M. xanthus *chromosome in single copy

We performed the site-directed mutagenesis of *mglA *using the PCR-overlap extension method [29]. To make each mutation, pairs of overlapping oligonucleotides, shown in Additional file 9: Table S1, were synthesized. The first round of PCR was done using each of two mutagenic oligonucleotides and each of two (flanking) oligonucleotides complementary either to the 5' or 3' ends of the *mglBA *operon, to amplify overlapping portions of this operon from pPLH325. Gel-purified PCR products were cloned into plasmid vector pCR2.1 Blunt TOPO and recombinant plasmids, otherwise isogenic with their parental plasmid, pPLH325, were recovered from Kan^R ^transformants of either *E. coli *JM107 or Top10. The presence of each mutation was confirmed by the analysis of the entire *mglBA *coding sequence by RFLP analysis and/or sequencing. These plasmids were introduced into the *M. xanthus *genome by homologous recombination.

### Examination of *mglA *transcription

*M. xanthus *strains were grown to a density of 5 × 10^8 ^cells/ml in CTPM medium at 32°C, and harvested by centrifugation. Total mRNA was harvested using Trizol reagent RNA extraction protocol (Invitrogen). Each sample was then treated with 6 units of DNaseI (Fermentas) for 45 min to remove any potential genomic DNA contaminants. 500 ng total RNA was used to produce cDNA using a Hexanucleotide Mix (Roche) as primer for the reaction using the Superscriptase II kit (Invitrogen). The resulting cDNA was diluted 1:25 or 1:1250 for probing target gene and 16s rRNA templates respectively. Primers were designed to amplify a region of 150 bp within each transcript, using the Power SYBR Green PCR 2× Master Mix kit (Applied Biosystems). qRT-PCR was performed using the Applied Biosystems 7900HT Real-Time system. The run was computer controlled by SDS 2.3 (Applied Biosystems). A no template control (NTC) was performed to provide a value for the background fluorescence present in a negative reaction. Three replicates for both the target and endogenous control were analyzed, and the target quantitation was normalized to the endogenous control for each replicate. The NTC was automatically subtracted from each RT-PCR reaction prior to averaging the replicates. The resulting data for each sample were calibrated to the WT expression levels and are shown as a relative quantity to the WT. A gene expression plot based on relative quantitation was generated using RQ Manager 1.2 (Applied Biosystems).

### Motility and developmental assays

Motility phenotypes of mutants were compared with that of the WT strain using swarm assays [58], by microscopic examinations of colony edges, and by time-lapse microscopy [59]. Swarm assays were performed in triplicate as described by Shi and Zusman [58]. Photomicrographs of the edges of isolated colonies were obtained using a Nikon FXA microscope with the 10× objective and captured by a Coolsnap Cf camera. Time-lapse microscopy was performed on CTPM medium with 1.5% Ultra-Pure agarose (Invitrogen) slabs. Cells were taken from mid-log phase liquid cultures and 50 μl of cell culture was pipetted onto the surface. Slabs were covered with a coverslip and incubated at 32° for 30 min prior to microscopic examination. For MC assays, 50 μl of mid-log phase cells were pipetted directly onto a slide inside a silicone gasket. After 20 min adherence at room temperature, the excess media were removed and the cells were overlaid with CTPM broth and 1% MC, (final concentration 0.5× and 0.5%, respectively). After a coverslip was placed, the slide was incubated at 32° for 30 min. Cells were photographed at 200× magnification, every 30 seconds for 30 min, yielding 61 time points for measurement.

Time-lapse data are based on 25 randomly chosen cells tracked for each strain and each condition. Strains that had fewer than 10% motile cells are classed as non-motile and their reversal rates were not determined. Motile cells were tracked in Metamorph, and their position data was used to generate velocity rates, but only reversing cells factored into cell reversal frequency by the Motility Macro v2.2 [60]. Cells were considered to reverse if they progressed one cell length then paused and moved in a new direction at least 110 degrees from the original direction of motion. Speeds are related in the text as the average of 25 cells ± the standard deviation.

To determine if mutations in the *mglA *gene affect the ability of *M. xanthus *to aggregate and sporulate, concentrated cells were plated onto TPM starvation medium as described [61]. Plates were incubated at 32° for 5 days, during which developing cells were monitored for aggregation, rippling, and fruiting body morphogenesis, using a Nikon SMZ-U stereomicroscope. To determine if rod-shaped *M. xanthus *cells had differentiated into heat-resistant spores, samples were scraped from starvation plates after 5 days, examined by microscopy for the presence of translucent, spherical spores, and titered after heat treatment at 50° on CTPM plates at 32^° ^to quantify spores capable of germination. In each of these experiments, strains DK1622 (WT) and DK6204 (Δ*mglBA*), MxH2419 and MxH2375 were used as controls and titrations were performed in triplicate.

### Immunoblot Analysis

Total cell lysate from three separate liquid cultures and Magic Mark (Invitrogen) standards were separated by SDS-PAGE with a 12.5% Tris-glycine gel. After electrophoresis, resolved proteins were transferred to a Polyscreen PVDF membrane (Perkin-Elmer). Blots were incubated with primary (polyclonal α-MglA 1:1000 dilution) and secondary (IR800-labeled Goat α-Mouse 1:2500 dilution; Rockland) antibodies. Blots were scanned using the 800 nm channel of a LiCor Odyssey Infrared Imager (LiCor Biosciences).

### Immunofluorescence analysis

*M. xanthus *strains were grown as previously described, then prepared as described [62] with a few alterations. Cells were fixed at 25 for 1 hr, and lysozyme was used at a concentration of 5 μg/ml for 15 min. After blocking overnight in 2% BSA (Sigma), slides were probed with α-MglA antibody at 1:200 and a 2° α-rabbit antibody labeled with Alexa fluor 488 (Rockland) at 1:400 dilution. Cells were visualized using the 60× objective lens of a Nikon 80i, with a YFP filter.

## Authors' contributions

PLH conceived the general outline of the experiments. SAF, NSB and PLH participated in planning and executing all molecular constructs and performed the assays. SAF performed the Immunofluorescence. SAF and PLH crafted the manuscript and constructed figures and movies. All authors have read and approved the final manuscript.

## Supplementary Material

Additional file 1 **Overlap of predicted MglA and experimentally derived Ras crystal structures**. This figure shows an overlay of the predicted MglA crystal structure with Ha-Ras to identify structures of particular interest. Areas of differences between the two structures are highlighted in this figure. Click here for file

Additional file 2 **Wild-type *Myxococcus xanthus *time-lapse in methylcellulose**. This movie shows the motility observed in WT *M. xanthus *in methylcellulose. Microscopy was performed as described in the Methods. Click here for file

Additional file 3 **Δ*mglBA M. xanthus *time-lapse in methylcellulose**. This movie shows the motility observed in *ΔmglBA M. xanthus *in methylcellulose, showing a decrease in gliding rates and the oscillating phenotype. Microscopy was performed as described in the Methods. Click here for file

Additional file 4 **MxH2410 *M. xanthus *time-lapse in methylcellulose**. This movie shows the gliding motility observed in the T26N mutant in methylcellulose, performed as described in the Methods. Click here for file

Additional file 5 **Double mutant *M. xanthus *time-lapse in methylcellulose**. This movie shows the phenotype of an A-S- double mutant in methylcellulose. Microscopy was performed as described in the Methods. Click here for file

Additional file 6 **Full length Western blot for MglA with internal loading control**. In order to discount the possibility that our inability to find MglA in several mutants was due to loading of the gel, we present this Western blot with loading control. Western analysis was performed as described in the Methods. Click here for file

Additional file 7 **Predicted RNA structure changes between WT *mgl *and Q82R *mgl *transcripts**. Using the RNAfold program, we analysed WT and Q82R *mgl *transcripts for differences in secondary structures. Click here for file

Additional file 8 **Western probing for MglA showing degradation during starvation-induced development**. This figure depicts a Western blot probing for MglA at different time points in development. Click here for file

Additional file 9 Table S1: This table contains all *M. xanthus *strains, *E. coli *strains, plasmids and oligonucleotides used in the construction of the mutants described in this study. Click here for file
