# Supplementary figures and images for: Effects of site-directed mutagenesis of mglA on motility and swarming of Myxococcus xanthus
Source: BMC Microbiol. 2010 Nov 18;10:295. doi: 10.1186/1471-2180-10-295 (PMC3000849; doi:10.1186/1471-2180-10-295)

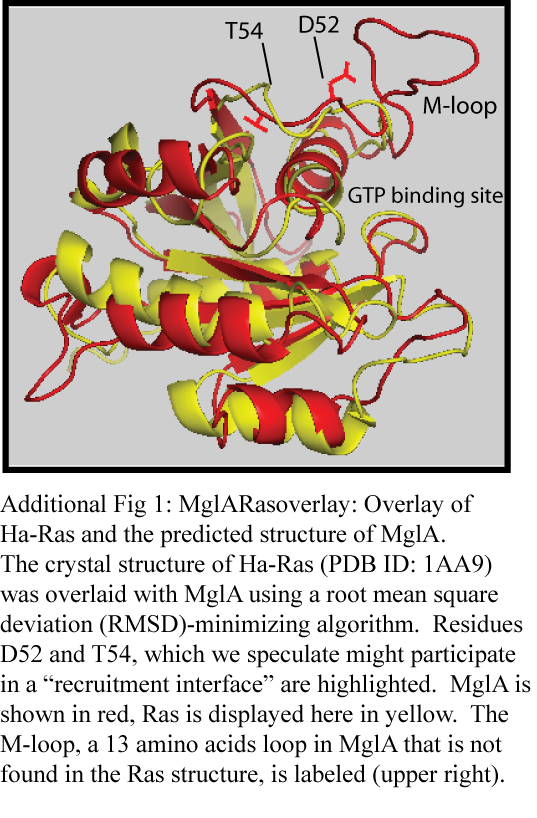

Supplement: Additional file 1 — Overlap of predicted MglA and experimentally derived Ras crystal structures. This figure shows an overlay of the predicted MglA crystal structure with Ha-Ras to identify structures of particular interest. Areas of differences between the two structures are highlighted in this figure. [file 1471-2180-10-295-S1.PNG]

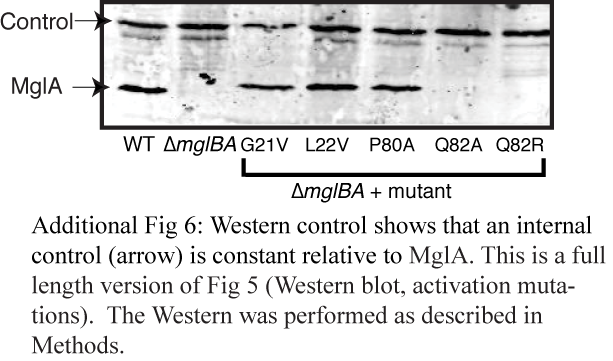

Supplement: Additional file 6 — Full length Western blot for MglA with internal loading control. In order to discount the possibility that our inability to find MglA in several mutants was due to loading of the gel, we present this Western blot with loading control. Western analysis was performed as described in the Methods. [file 1471-2180-10-295-S6.PNG]

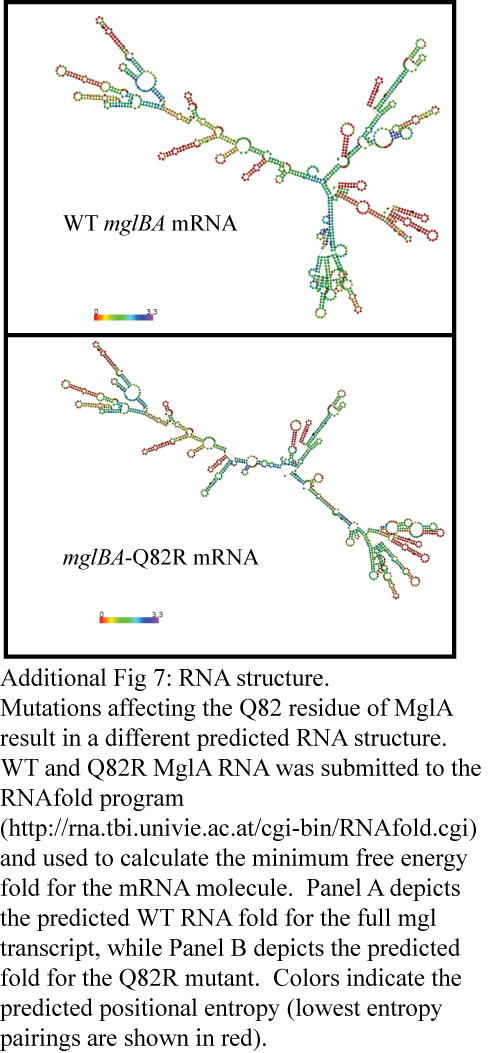

Supplement: Additional file 7 — Predicted RNA structure changes between WT mgl and Q82R mgl transcripts. Using the RNAfold program, we analysed WT and Q82R mgl transcripts for differences in secondary structures. [file 1471-2180-10-295-S7.PNG]

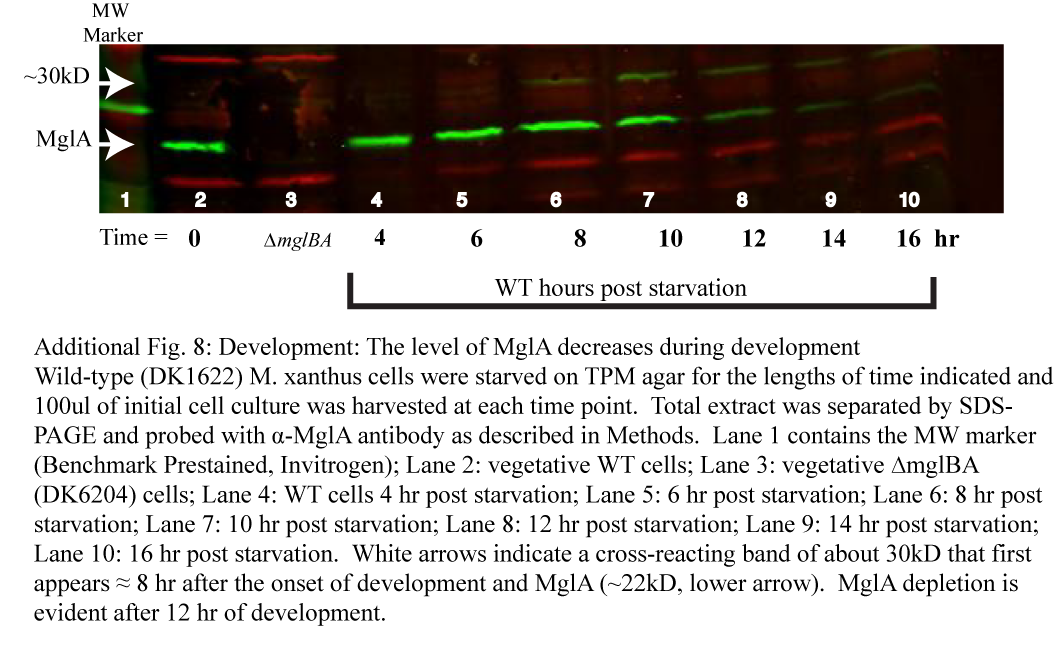

Supplement: Additional file 8 — Western probing for MglA showing degradation during starvation-induced development. This figure depicts a Western blot probing for MglA at different time points in development. [file 1471-2180-10-295-S8.PNG]
