# Supplementary material for: Effects of site-directed mutagenesis of mglA on motility and swarming of Myxococcus xanthus
Source: BMC Microbiol. 2010 Nov 18;10:295. doi: 10.1186/1471-2180-10-295 (PMC3000849; doi:10.1186/1471-2180-10-295)
Supplement: Additional file 9 — Table S1: This table contains all M. xanthus strains, E. coli strains, plasmids and oligonucleotides used in the construction of the mutants described in this study. [file 1471-2180-10-295-S9.DOC]

# Additional Table 1. Strains and molecular reagents

| Strain Name | Genotype | Phenotype | Construction | Reference |
| --- | --- | --- | --- | --- |
| DK1622 | WT | A+S+ motility Kan^S^ |  | [5] |
| DK6204 | Δ*mglBA* | non-motile Kan^S^ | 834bp *Bal*I deletion in *mglBA* | [23] |
| MxH2247 | Δ*mglBA* pNB16 | Kan^R^ MglA-T78A | pNB16 in DK6204 | This Work |
| MxH2248 | Δ*mglBA* pNB17 | Kan^R^ MglA-T78S | pNB17 in DK6204 | This Work |
| MxH2278 | WT pKV51 | Kan^R^ MglA-L124K | pKV51 in DK1622 | This Work |
| MxH2279 | Δ*mglBA* pKV51 | Kan^R^ MglA-L124K | pKV51 in DK6204 | This Work |
| MxH2319 | Δ*mglBA* pNB89 | Kan^R^ MglA-Q82R | pNB89 in DK6204 | This Work |
| MxH2320 | Δ*mglBA* pSF4 | Kan^R^ MglA-Q82A | pSF4 in DK6204 | This Work |
| MxH2336 | WT pNB91 | Kan^R^ MglA-N141A | pNB91 in DK1622 | This Work |
| MxH2337 | WT pNB64 | Kan^R^ MglA-L117-120A | pNB64 in DK1622 | This Work |
| MxH2338 | Δ*mglBA* pNB91 | Kan^R^ MglA-N141A | pNB91 in DK6204 | This Work |
| MxH2339 | Δ*mglBA* pNB64 | Kan^R^ MglA-L117/120A | pNB64 in DK6204 | This Work |
| MxH2356 | WT pNB88 | Kan^R^ MglA-P80A | pNB88 in DK1622 | This Work |
| MxH2357 | Δ*mglBA* pNB88 | Kan^R^ MglA-P80A | pNB88 in DK6204 | This Work |
| MxH2358 | WT pNB85 | Kan^R^ MglA-L22V | pNB85 in DK1622 | This Work |
| MxH2359 | Δ*mglBA* pNB85 | Kan^R^ MglA-L22V | pNB85 in DK6204 | This Work |
| MxH2360 | WT pNB84 | Kan^R^ MglA-G21V | pNB84 in DK1622 | This Work |
| MxH2361 | Δ*mglBA* pNB84 | Kan^R^ MglA-G21V | pNB84 in DK6204 | This Work |
| MxH2364 | WT pNB92 | Kan^R^ MglA-K142A | pNB92 in DK1622 | This Work |
| MxH2365 | Δ*mglBA* pNB92 | Kan^R^ MglA-K142A | pNB92 in DK6204 | This Work |
| MxH2366 | WT pNB93 | Kan^R^ MglA-D144A | pNB93 in DK1622 | This Work |
| MxH2367 | Δ*mglBA* pNB93 | Kan^R^ MglA-D144A | pNB93 in DK6204 | This Work |
| MxH2368 | WT pNB89 | Kan^R^ MglA-Q82R | pNB89 in DK1622 | This Work |
| MxH2375 | WT pKD100 | Kan^R^ MglBA merodiploid | pKD100 in DK1622 | This Work |
| MxH2390 | Δ*mglBA* pKD108 | Kan^R^ MglB | pKD108 in DK6204 | This Work |
| MxH2391 | WT pKD108 | Kan^R^ MglB merodiploid | pKD108 in DK1622 | This Work |
| MxH2404 | WT pSF4 | Kan^R^ MglA-Q82A | pSF4 in DK1622 | This Work |
| MxH2405 | WT pJP17 | Kan^R^ MglA-T54A | pJP17 in DK1622 | This Work |
| MxH2406 | Δ*mglBA* pJP17 | Kan^R^ MglA-T54A | pJP17 in DK6204 | This Work |
| MxH2407 | WT pJP18 | Kan^R^ MglA-D52A | pJP18 in DK1622 | This Work |
| MxH2408 | Δ*mglBA* pJP18 | Kan^R^ MglA-D52A | pJP18 in DK6204 | This Work |
| MxH2409 | WT pSF1 | Kan^R^ MglA-T26N | pSF1 in DK1622 | This Work |
| MxH2410 | Δ*mglBA* pSF1 | Kan^R^ MglA-T26N | pSF1 in DK6204 | This Work |
| MxH2419 | Δ*mglBA* pKD100 | Kan^R^ MglBA | pKD100 in DK6204 | This Work |
| MxH2425 | WT pNB16 | Kan^R^ MglA-T78A | pNB16 in DK1622 | This Work |
| MxH2426 | WT pNB17 | Kan^R^ MglA-T78S | pNB17 in DK1622 | This Work |
| MxH2428 | WT pPLH113 | Kan^R^ MglA-T78D | pNB9 in DK1622 | This Work |
| MxH2429 | WT pSF3 | Kan^R^ MglA-K25A | pSF3 in DK1622 | This Work |
| MxH2430 | Δ*mglBA* pSF3 | Kan^R^ MglA-K25A | pSF3 in DK6204 | This Work |
| MxH2431 | WT pSF2 | Kan^R^ MglA-G19A | pSF2 in DK1622 | This Work |
| MxH2432 | Δ*mglBA* pPLH113 | Kan^R^ MglA-T78D | pNB9 in DK6204 | This Work |
| MxH2445 | Δ*mglBA* pSF2 | Kan^R^ MglA-G19A | pSF2 in DK6204 | This Work |

| Plasmid | Relevant Features | Construction | Reference |
| --- | --- | --- | --- |
| pCR2.1 | *npt*II | pCR II Blunt Topo | Invitrogen |
| pJP17 | *mglB mglA*-T54A *npt*II | PCR378 cloned into pCR2.1 | This Work |
| pJP18 | *mglB mglA-*D52A *npt*II | PCR375 cloned into pCR2.1 | This Work |
| pKD100 | *mglB mglA npt*II | PCR520 cloned into pCR2.1 | This Work |
| pKD108 | *mglB npt*II | PCR522 cloned into pCR2.1 | This Work |
| pKV51 | *mglB mglA-*L124K *npt*II | PCR511 cloned into pCR2.1 | This Work |
| pPLH113 | *mglB mglA*-T78D *npt*II | *Hind*III-*Bam*HI digest of PCR180 ligated with *Hin*dIII-*Bam*HI pBGS18 | This Work |
| pPLH325 | *mglB mglA nptII* | Chromosomal *Pst*I fragment containing the *mglBA* operon | [23] |
| pNB16 | *mglB mglA-T78A npt*II | PCR320 cloned into pCR2.1 | This Work |
| pNB17 | *mglB mglA*-T78S *npt*II | PCR351 cloned into pCR2.1 | This Work |
| pNB64 | *mglB mglA-*L117/120A *npt*II | PCR372 cloned into pCR2.1 | This Work |
| pNB84 | *mglB mglA*-G21V-His6 *npt*II | PCR512 cloned into pCR2.1 | This Work |
| pNB85 | *mglB mglA*-L22V-His6 *npt*II | PCR513 cloned into pCR2.1 | This Work |
| pNB88 | *mglB mglA*-P80A-His6 *npt*II | PCR357 cloned into pCR2.1 | This Work |
| pNB89 | *mglB mglA-*Q82R-His6 *npt*II | PCR366 cloned into pCR2.1 | This Work |
| pNB91 | *mglB mglA-*N141A *npt*II | PCR369 cloned into pCR2.1 | This Work |
| pNB92 | *mglB mglA-*K142A-His6 *npt*II | PCR360 cloned into pCR2.1 | This Work |
| pNB93 | *mglB mglA-*D144A-His6 *npt*II | PCR354 cloned into pCR2.1 | This Work |
| pSF1 | *mglB mglA-*T26N *npt*II | PCR502 cloned into pCR2.1 | This Work |
| pSF2 | *mglB mglA-*G19A *npt*II | PCR505 cloned into pCR2.1 | This Work |
| pSF3 | *mglB mglA-*K25A *npt*II | PCR508 cloned into pCR2.1 | This Work |
| pSF4 | *mglB mglA-*Q82A *npt*II | PCR363 cloned into pCR2.1 | This Work |

| PCR reaction | primers used | template | reaction product |
| --- | --- | --- | --- |
| PCR177 | *49x103* | pPLH325 |  |
| PCR178 | *102x94* | pPLH325 |  |
| PCR180 | *49x94* | PCR177 and 178 overlap | T78D mutant |
| PCR317 | *49x277* | pPLH325 |  |
| PCR318 | *276x94* | pPLH325 |  |
| PCR320 | *49x94* | PCR317 and 318 overlap | T78A mutant |
| PCR343 | *49x279* | pPLH325 |  |
| PCR344 | *278x94* | pPLH325 |  |
| PCR351 | *49x94* | PCR343 and 344 overlap | T78S mutant |
| PCR352 | *546x87* | pPLH325 |  |
| PCR353 | *86x522* | pPLH325 |  |
| PCR354 | *546x522* | PCR352 and 353 overlap | D144A mutant |
| PCR355 | *49x350* | pPLH325 |  |
| PCR356 | *349x522* | pPLH325 |  |
| PCR357 | *49x522* | PCR355 and 356 overlap | P80A mutant |
| PCR358 | *546x352* | pPLH325 |  |
| PCR359 | *351x522* | pPLH325 |  |
| PCR360 | *546x522* | PCR358 and 359 overlap | K142A mutant |
| PCR361 | *546x362* | pPLH325 |  |
| PCR362 | *361x93* | pPLH325 |  |
| PCR363 | *546x93* | PCR361 and 362 overlap | Q82A mutant |
| PCR364 | *546x368* | pPLH325 |  |
| PCR365 | *367x522* | pPLH325 |  |
| PCR366 | *546x522* | PCR364 and 365 overlap | Q82R mutant |
| PCR367 | *546x424* | pPLH325 |  |
| PCR368 | *423x522* | pPLH325 |  |
| PCR369 | *546x522* | PCR367 and 368 overlap | N141A mutant |
| PCR370 | *49x439* | pPLH325 |  |
| PCR371 | *438x522* | pPLH325 |  |
| PCR372 | *49x522* | PCR 370 and 371 overlap | L117/120A mutant |
| PCR373 | *546x539* | pPLH325 |  |
| PCR374 | *538x522* | pPLH325 |  |
| PCR375 | *546x522* | PCR373 and 374 overlap | D52A mutant |
| PCR376 | *546x545* | pPLH325 |  |
| PCR377 | *544x522* | pPLH325 |  |
| PCR378 | *546x522* | PCR376 and 377 overlap | T54A mutant |
| PCR500 | *546x627* | pPLH325 |  |
| PCR501 | *626x93* | pPLH325 |  |
| PCR502 | *546x93* | PCR500 and 501 overlap | T26N mutant |
| PCR503 | *546x657* | pPLH325 |  |
| PCR504 | *656x93* | pPLH325 |  |
| PCR505 | *546x93* | PCR503 and 504 overlap | G19A mutant |
| PCR506 | *546x659* | pPLH325 |  |
| PCR507 | *658x93* | pPLH325 |  |
| PCR508 | *546x93* | PCR506 and 507 overlap | K25A mutant |
| PCR509 | *49x48* | pPLH325 |  |
| PCR510 | *47x93* | pPLH325 |  |
| PCR511 | *49x93* | PCR509 and 510 overlap | L124K mutant |
| PCR512 | *49x522* | pPLH325 | G21V |
| PCR513 | *129* | PCR512 |  |
| PCR514 | *49x522* | pPLH325 | L22V |
| PCR515 | *130s* | PCR514 |  |
| PCR520 | *49x93* | WT chromosomal | *mglBA* |
| PCR522 | *546x221* | WT chromosomal | *mglB* |

| Primer | Sequence |  |
| --- | --- | --- |
| 47 | TGCTCCCGC**CTT**GTTGATGCGGAGGTTCTC | L124K R |
| 48 | GCATCAAAC**AAG**GCGGAGCAGGGCTACGAC | L124K F |
| 49 | ATCACAAGCTTAAGAGTCAGGCCCC | 5' end of operon |
| 86 | ACAAGCGCG**CGC**TGCCCAACGCGGTGACG | D144A F |
| 87 | TTGGGCA**GCG**CGCGCTTGTTGTACTGGAT | D144A R |
| 93 | ATCGAATTCAGCGGATGCGGCGGGCT | 3' end of operon EcoRI site |
| 94 | GGAATTCGTCACGCGTGATGATGGTGGTGATGGCCACCCTTCTTGAGCTCG | MglA-His |
| 102 | CCTGTAC**GAC**GTGCCCGGTCAG | T78D F |
| 103 | CCGGGCAC**GTC**GTACAGGTGCAACA | T78D R |
| 129 | TACGGGCCC**GTG**CTCTGCGGGAAGACGACCAA | G21V |
| 130 | TAGGGGCCCGGG**GTC**TGCGGGAAGACGACCAA | L22V |
| 221 | GGAATTCCTCGCTGAAGAGGTTGTCGA | 3’ *mglB* |
| 276 | CCACCTGTAC**GCG**GTGCCCGGTCAGGTCTTCTACGACGCCA | T78A F |
| 277 | CCGGGCACC**GCG**TACAGGTGGAAGCGCGTCTTGAAGCCG | T78A R |
| 278 | CCACCTGTAC**TCG**GTGCCCGGTCAGGTCTT CTACGACGCCA | T78S F |
| 279 | CCGGGCAC**CGA**GTACAGGTGGAAGCGCGTCTTGAAGCCG | T78S R |
| 349 | GGACATGTGCCAC**CGG**CCAGTCCAG | P80A F |
| 350 | GACCTGACC**GGC**CACCGTGTACAGG | P80A R |
| 351 | CTGAACAAGATTCGTACGTCATCCAGTACCAC**GCG**CGCGACCTGCCCA | K142A F |
| 352 | CAGGTCGCG**CGC**GTTGTACTGGATG | K142A R |
| 361 | CCCGGT**GCG**GTCTTCTACGACGCCAGCCGCAAGCTCATCCTC | Q82A F |
| 362 | GTCGTAGAAGAC**CGC**ACCGGGCACCGTGTACAGGTG | Q82A R |
| 367 | GCCCGGT**CGG**GTCTTCTACGACGCCAGCCGCAAGCTCATCCTC | Q82R F |
| 368 | GCGTCGTAGAAGAC**CCG**ACCGGGCACCGTGTACAGGT | Q82R R |
| 423 | TCCAGTAC**GCC**AAGCGCGACCTGCCAACGCGGTG | N141 F |
| 424 | TCGCGCTT**GGC**GTACTGGATGACGTACGGAATCTT | N141 R |
| 438 | GGAGTCG**GC**CGAGAAC**GC**CCGCATCAACCTTGCGGAGCAGGGCTACGA | L117A/120A F |
| 439 | GATGCGG**GC**GTTCTCG**GC**CGACTCCATGTTCGCTTCCATCGCTTG | L117A/120A R |
| 522 | TCAGTGGTGGTGGTGGTGGTGACCACCCTTCTTGAGCTCGGTGAG | MglA-His |
| 538 | CCCTCTCCACGGAGACGG**CCC**GCACGCTCTTCTTCGACTTCC | D52A F |
| 539 | GGAAGTCGAAGAAGAGCGTGC**GGG**CCGTCTCCGTGGAGAGGG | D52A R |
| 544 | CCCTCTCCACGGAGACGGACC**GCG**CGCTCTTCTTCGACTTCC | T54A F |
| 545 | GGAAGTCGAAGAAGAG**CGC**GCGGTCCGTCTCCGTGGAGAGGG | T54A R |
| 546 | AAGCTTGCGGAGTATAGAGAAAGCCGGGAAGGCCCC | 5' end of operon |
| 626 | TACGGGCCCGGGCTCTGCGGGAAG**AAC**ACCCTTCAGTACATCTACAA | T26N F |
| 627 | TTGTAGATGTACTGAAGGGT**GTT**CTTCCCGCAGAGCCCGGGCCCGTA | T26N R |
| 656 | TCTATTAC**GCG**CCCGGGCTCTGCGGGAAGACGACCAACC | G19A F |
| 657 | CCCGCAGAGCCCGG**GCG**CGTAATAGACAATCTTGCAGTTGATTTC | G19A R |
| 658 | CTGCGGG**GCG**ACGACCAACCTTCAGTACATCTACAACAAGACCGCCG | K25A F |
| 659 | GTACTGAAGGTTGGTCGT**CGC**CCCGCAGAGCCCGGGCCCGTAATAGAC | K25A R |
